# Using expected sequence features to improve basecalling accuracy of amplicon pyrosequencing data

**DOI:** 10.1186/s12859-016-1032-7

**Published:** 2016-04-22

**Authors:** Thomas S. Rask, Bent Petersen, Donald S. Chen, Karen P. Day, Anders Gorm Pedersen

**Affiliations:** Department of Systems Biology, Center for Biological Sequence Analysis, Technical University of Denmark, Building 208, Kongens Lyngby, DK-2800 Denmark; Division of Medical Parasitology, Department of Microbiology, New York University Langone Medical Center, 341 East 25th Street, New York, NY 10010 USA; School of Biosciences, The University of Melbourne, Parkville, Victoria 3010 Australia

**Keywords:** Bayesian methods, Basecalling, Amplicon sequencing

## Abstract

**Background:**

Amplicon pyrosequencing targets a known genetic region and thus inherently produces reads highly anticipated to have certain features, such as conserved nucleotide sequence, and in the case of protein coding DNA, an open reading frame. Pyrosequencing errors, consisting mainly of nucleotide insertions and deletions, are on the other hand likely to disrupt open reading frames. Such an inverse relationship between errors and expectation based on prior knowledge can be used advantageously to guide the process known as basecalling, i.e. the inference of nucleotide sequence from raw sequencing data.

**Results:**

The new basecalling method described here, named Multipass, implements a probabilistic framework for working with the raw flowgrams obtained by pyrosequencing. For each sequence variant Multipass calculates the likelihood and nucleotide sequence of several most likely sequences given the flowgram data. This probabilistic approach enables integration of basecalling into a larger model where other parameters can be incorporated, such as the likelihood for observing a full-length open reading frame at the targeted region. We apply the method to 454 amplicon pyrosequencing data obtained from a malaria virulence gene family, where Multipass generates 20 % more error-free sequences than current state of the art methods, and provides sequence characteristics that allow generation of a set of high confidence error-free sequences.

**Conclusions:**

This novel method can be used to increase accuracy of existing and future amplicon sequencing data, particularly where extensive prior knowledge is available about the obtained sequences, for example in analysis of the immunoglobulin VDJ region where Multipass can be combined with a model for the known recombining germline genes. Multipass is available for Roche 454 data at http://www.cbs.dtu.dk/services/MultiPass-1.0, and the concept can potentially be implemented for other sequencing technologies as well.

**Electronic supplementary material:**

The online version of this article (doi:10.1186/s12859-016-1032-7) contains supplementary material, which is available to authorized users.

## Background

DNA sequencing during the last three decades has been dominated by the Sanger method [1], for which the main type of sequencing error is nucleotide substitutions [2]. Recently, many new massively parallelized sequencing methods have become available, amongst those pyrosequencing, implemented in a commercial product by Roche 454 (Roche 454 Sequencing, http://www.454.com/) [3]. 454 pyrosequencing is distinguished from other available high throughput methods by its long read length, as well as the main error type inherent to the method which is insertions and deletions (indels), occurring at a rate around 1 % [4].

The initial step in 454 pyrosequencing is attachment of sequencing adaptors to template DNA, known as library preparation, for example by shearing of genomic DNA and subsequent ligation of adaptors. Emulsion PCR is then performed with primer coated capture beads in a specific concentration ratio so a single template library sequence is clonally amplified on the surface of one bead. Each bead is loaded in one of 1.6 million wells on a picotiter plate (PTP) where DNA synthesis takes place. The PTP is exposed to flows of PCR reagents across the open wells with one nucleotide type at a time. A typical sequencing run consists of 200 cycles of four flows (TACG). Each flow results in a number of incorporated nucleotides matching the template, which releases a proportional amount of pyrophosphate that through enzymatic reaction gives rise to a light signal in each well, where the intensity indicates the number of nucleotides incorporated in the read [3]. The measured raw light intensities from each flow are normalized and corrected for artifacts, amongst other pertaining to well position on the PTP and gradual build-up of asynchrony along the read. Furthermore, low quality sequence is identified and removed using various strategies [5]. Hence, each read is given as a sequence of 800 pre-processed light intensities, known as a flowgram, where each flow value gives the length of a homopolymer (HP) in the read.

Basecalling is the process of inferring the DNA sequence that gave rise to a given set of sequencing data. To achieve this in 454 pyrosequencing, bases can be called directly from each flowgram by rounding flow values to the nearest integer. This process, however, throws away information that can be useful for error correction, if several reads exist from identical templates, which is often the case. The AmpliconNoise [6] package is a widely used package for 454 basecalling, which initially clusters and aligns flowgrams from identical templates, and uses all flowgrams in the alignment to call the most likely nucleotide sequence.

A now widely used technique for measurement of bio- and genetic diversity is amplicon sequencing, where a variable target region is PCR amplified using region specific PCR primers designed with sequencing adaptor extensions allowing direct sequencing of the amplicon. 454 pyrosequencing is often used for amplicon sequencing due to simple adaptor design and long reads. The fact that the target sequence is known to some extent gives an expectation about which features the amplicon sequences should possess. For example a region of a gene encoding a protein with an important function in the organism could be highly expected to have a full-length open reading frame (FRF).

We present here a probabilistic framework for pyrosequencing basecalling, named Multipass since it passes on multiple sequence alternatives, as well as their likelihood given the flowgram data, for integration in a larger model. Using Multipass we demonstrate how the expectation of a full-length open reading frame in amplicon sequences can be used in such a model, to improve sequence quality. The method can furthermore take advantage of bi-directional pyrosequencing data, which assures quality sequence in both ends of the read. Finally, Multipass reports useful parameters for estimation of the quality of the called sequence, which allows creation of a high confidence error-free sequence set.

## Methods

To exemplify the use of the novel basecalling method, we applied it to amplicon pyrosequencing data obtained from 12 samples composed of the *Plasmodium falciparum* laboratory reference strains 3D7, HB3, and DD2 (Additional file 1: Table S1). Three main steps of data processing were performed: calculation of the most likely basecalls from the raw sequencing data using Multipass; integration of the basecalls in a probabilistic model that takes prior knowledge into account to improve basecalling accuracy; and finally definition of a subset of high quality sequences.

### Pyrosequencing

#### *Var* gene DBLα PCR amplification for pyrosequencing

DNA from *Plasmodium falciparum* reference strain laboratory cultures was extracted using the DNeasy Blood and Tissue kit (Qiagen, France) according to the manufacturer’s recommendations, and eluted in 100 μL of elution buffer per 200 μL of whole blood. We performed PCR amplification of the DBLα domain of the *var* genes using fusion primers for multiplexed 454 Titanium sequencing. We coupled template-specific degenerated primer sequences targeting homology block 2 and 3 [7, 8]: DBLαAF, 5’-GCACGMAGTTTYGC-3’ and DBLαBR, 5’-GCCCATTCSTCGAACCA- 3’. Specifically, forward and reverse primers were designed by adding GS FLX Titanium Primer sequence and 10 bp multiplex identifier (MID) tags published by Roche (Roche 454 Sequencing Technical Bulletin No. 013-2009; 454 Sequencing Technical Bulletin No. 005-2009). These MID’s have been engineered to avoid misassignment of reads and they are tolerant to several errors. Every 40 μL reaction mix was composed of 3 μL of each primer (10 μM), 1.4 μL dNTP mix (2 mM), 4 μL buffer 5X, 2 μL of MgCl2, 0.6 μL Taq polymerase (Promega, GoTaq polymerase, 5UI/μL) and 1 μL of isolate. Amplifications were carried out in a thermal cycler using the following reaction conditions: 30 cycles of 95 °C for 40 s, 49 °C for 1 min 30 sec, 65 °C for 1 min 30 sec, and a final extension step of 65 °C for 10 min. These tagged primers were validated for amplification of sequences of the appropriate length using *P. falciparum* 3D7 genomic DNA. PCR amplification was confirmed visually by nucleic acid staining (EZ VISION™ DNA Dye, Ambresco) followed by gel electrophoresis (2 % agarose in 0.5x TBE buffer) demonstrating a band of the appropriate size (~477 bp). Negative controls (no template) were performed for quality assurance.

#### Amplicon library preparation and 454 Titanium sequencing

The PCR products were first purified using solid-phase reversible immobilization (SPRI) method (Agencourt, AMPure XP). Then, PCR amplicon concentrations were measured using the Quant-iT PicoGreen dsDNA kit per manufacturer’s instructions (Invitrogen). Known concentrations of control DNA were prepared as directed by the Roche Technical Bulletin (454 Sequencing Technical Bulletin No. 005-2009). We assayed fluorescence intensity using a Perkin-Elmer VICTOR X3 multilabel plate reader, with fluorescein excitation wavelength of ~480 nm and emission of ~520 nm wavelength. We prepared PCR amplicon library pools, each containing equimolar amounts of the PCR amplicons with unique MID tags. These pools were sequenced in forward and reverse directions on segregated regions using 454 GS FLX Titanium chemistry (Roche). Sequencing was performed by Seqwright Genomics (Houston, TX, USA) and New York University Genome Technology Center (New York, NY, USA).

### Sequence data processing

#### Demultiplexing and flowgram clustering

MID-tags and primers were identified (exact match) and trimmed off the flowgrams, reverse reads were reverse complemented, and a dat-file with the resulting flowgrams was created for each MID, using BioPython v1.57. Flowgram clustering was performed using the AmpliconNoise package v1.25 [9]. This method thus takes advantage of bi-directional sequencing, as reads in both directions are included in each cluster, so the forward reads will give high quality in the 5’-end of the target sequence, and the reverse reads will improve the 3’-end quality. A future improvement of the method could be to weight reads of each direction differently in the sequence ends during basecalling.

#### Homopolymer flow distributions in 454 pyrosequencing

The challenge in 454 basecalling is to estimate homopolymer lengths from flow signals. In the probabilistic framework employed in this paper, an important step in accomplishing this, is to compare observed flow values to empirical probability distributions that indicate how likely different possible flow values are for any given homopolymer length. Such flow value distributions observed for different homopolymer lengths have previously been described for 0 to 5 nucleotides, and an extrapolation for longer homopolymers was suggested (normal distributions with *μ = h* and *σ =* 0.06856 *h +* 0.03494, where *h* is the homopolymer length) [10]. When we performed maximum likelihood fitting of normal distributions to flow signal distributions from homopolymers longer than 5 nucleotides present in the *Pf* DBLα-tag reference sequences, we found that these homopolymers gave lower flow signals than what was expected from the Balzer extrapolation, and that normal distributions shifted towards zero described the 454 data more accurately (Additional file 1: Figure S1). Homopolymers of length ≥9 were better described by normal distributions with mean according to the equation *μ =* 0.525 *h +* 4.177, and variance as suggested in [10].

Since the distant tails of (log-)normal distributions do not describe empirical flow distributions well, and due to the presence of noise in the flow signals, a minimal probability *P(s|x ≤ h < x + 0.1) = 10*^*-10*^ was employed for any given flow interval of length 0.1 flow units.

#### Multiple flowgram alignment

We developed a program for pairwise flowgram alignment in the language C based on the Needleman–Wunsch global alignment algorithm. For this purpose we need a scoring matrix that, for each possible pair of flow values, assigns a score indicating how likely it is that the two flows should be aligned. In analogy to sequence alignment, the scoring matrix we employ consists of log-odds scores, where the numerator of the odds-ratio is the probability that a pair of flow signals originate from the same homopolymer, while the denominator is the probability of observing the flow signals irrespective of their origin. The details of how to compute these values are as follows: We have two flow signals *s*_*1*_, *s*_*2*_ of flow type *N*_*1*_, *N*_*2*_, respectively (where “flow type” means the nucleotide used for the flow). These flow signals originated from homopolymers of length *h*_*1*,_*h*_*2*_ and type *n*_*1*_, *n*_*2*_, respectively. The log-odds score is then calculated as *S*(*s*_*1*_*,s*_*2*_*,N*_*1*_*,N*_*2*_) *=* log(*OR*). The odds-ratio can be found as follows:

1 $$ \begin{array}{ll} OR& =\frac{P\left({h}_1={h}_2,{n}_1={n}_2,{s}_1,{s}_2,{N}_1,{N}_2\right)}{P\left({s}_1,{s}_2,{N}_1,{N}_2\right)}\\ {}& =P\left({h}_1={h}_2,{n}_1={n}_2|{s}_1,{s}_2,{N}_1,{N}_2\right)\\ {}& =P\left({h}_1={h}_2|{s}_1,{s}_2\right)P\left({n}_1={n}_2|{N}_1,{N}_2\right)\\ {}& =P\left({n}_1={n}_2|{N}_1,{N}_2\right)\sum_{i=0}^{h_{max}}P\left({h}_1=i|{s}_1\right)P\left({h}_2=i|{s}_2\right)\end{array} $$

In this derivation we have used the definition of conditional probability, the assumption that homopolymer lengths are independent of flow types, and the law of total probability. *h*_*max*_ was set to 30. Since it is nearly impossible that a flow signal originates from a nucleotide type different from the flow type, we defined:

2 $$ P\left({n}_1={n}_2|{N}_1,{N}_2\right)=\Big\{\begin{array}{ll}{10}^{-100},& \mathrm{if}\kern0.5em {N}_1\ne {N}_2\\ {}1-{10}^{-100},& \mathrm{if}\kern0.5em {N}_1={N}_2\end{array}\operatorname{} $$

Using Bayes’ theorem, the probability of a homopolymer length *h* given a signal *s* was calculated as:

3 $$ P\left(h|s\right)=\frac{P\left(s|h\right)P(h)}{\sum_{j=0}^{h_{max}}P\left(s|j\right)P(j)} $$

where *P*(*h*) is the probability of encountering a homopolymer of length *h* in a flowgram, and *P*(*s*|*h*) was determined from the homopolymer distributions (Additional file 1: Figure S1, and Additional file 2). A gap penalty of log(10^-10^) was used. For each pairwise alignment, a consensus flowgram was calculated with a mean flow for each alignment position. Using such pairwise alignment, we developed a script that performs multiple flowgram alignment, by iteratively aligning the two most similar flowgram profiles, the same approach as used by the multiple sequence alignment program Clustal [11].

#### Basecalling from multiple flowgram alignments

The likelihood of a nucleotide sequence being the correctly basecalled sequence (CBS) given the flow values of the flowgram alignment, was calculated as:

4 $$ P\left(CBS|flows\right)=\prod_{l=0}^LP\left({h}_l|{S}_l\right) $$

where *L* is the flowgram alignment length, *h*_*l*_ is the homopolymer length at alignment position *l*, and *S*_*l*_ represents all flow values at position *l*. The probability of a given homopolymer length *h*, at position *l* with flow values *S*_*l*_, was calculated using Bayes’ theorem as:

5 $$ \begin{array}{ll}P\left(h|{S}_l\right)& =\frac{P\left({S}_l|h\right)P(h)}{\sum_{j=0}^{h_{max}}P\left({S}_l|j\right)P(j)}\\ {}& =\frac{P(h){\prod}_{m=1}^MP\left({s}_{l,m}|h\right)}{\sum_{j=0}^{h_{max}}\left[P(j){\prod}_{m=1}^MP\left({s}_{l,m}|j\right)\right]}\end{array} $$

where *M* is the number of flowgrams in the alignment, and *s*_*l,m*_ is the flow value at position *l* in sequence *m*.

The probability of a flow value *s* given the homopolymer length *h*, *P*(*s*_*l,m*_*|h*), was derived from the normal and log-normal distributions obtained by fitting to empirical data as described above (Additional file 1: Figure S1).

The *N* most likely nucleotide sequences were thus calculated from each aligned flowgram cluster using Equation 4. The state of the expected sequence feature was then determined in these nucleotide sequences, i.e. the presence or absence of a forward full-length open reading frame was established, or sequences were scored by alignment to a profile hidden Markov model (see the two following sections). Finally, the probability that a sequence was correctly basecalled was calculated for each of the *N* most likely sequences. For a sequence to be correctly basecalled, it both has to be so in the sense that it gave rise to the sequencing flows (*CBS*_*flows*_), as well as in the sense that it came from the amplified genomic target region and therefore has certain sequence features (*CBS*_*feature*_). Hence, the most likely correctly basecalled sequence was selected as the one with the maximal joint probability:

6 $$ \begin{array}{l}P\left(CBS\left| flows, feature\right.\right)\\ {}\kern2.16em =P\left(CB{S}_{flows},CB{S}_{feature}\left| flows, feature\right.\right)\\ {}\kern2.16em =P\left(CB{S}_{flows}\left| flows\right.\right)P\left(CB{S}_{feature}\left| feature\right.\right)\end{array} $$

where it is assumed that *CBS*_*flows*_ and *CBS*_*feature*_ are conditionally independent given *flows*,*feature*, that *CBS*_*flows*_ and *feature* are conditionally independent given *flows*, and that *CBS*_*feature*_ and *flows* are conditionally independent given *feature. P*(*CBS*_*feature*_|*feature*) is the probability that the sequence is correctly basecalled given the state of the feature in the sequence. In this study *P*(*CBS*_*feature*_|*feature*) equals either *P*(*CBS*|*FRF*) or *P*(*CBS*|*S*_*HMM*_).

#### Calculating sequence likelihoods using full-length open reading frame as feature

A sequence was considered to have a full-length open reading frame if any of the three forward reading frames lacked a stop codon. The probability of each of the *N* = 10 most likely basecalls being a true DBLα sequence given the presence of a full-length ORF was calculated using Bayes theorem:

7 $$ P\left(CBS\left|FRF\right.\right)=\frac{P\left(FRF\left|CBS\right.\right)P(CBS)}{P(FRF)} $$

where the likelihood of presence of a FRF given a true DBLα sequence *P*(*FRF*|*CBS*) = 0.9979 was determined as the frequency of FRFs in DBLα-tags from 227 Illumina sequenced *Plasmodium falciparum* genomes [12]. 6799 out of 6813 DBLα-tags had a full-length open reading frame, however the actual frequency may be even higher since we can not exclude the possibility of Illumina sequencing errors, and a random SNP is more likely to disrupt than create a FRF. The prior likelihood of randomly choosing the true DBLα sequence was set to *P*(*CBS*) ~ 1/*N =* 0.1 since we are selecting one out of ten sequences and the correct basecall of nearly all control sequences was found among the ten most likely basecalls. Finally, the normalization likelihood of any basecall having a full-length open reading frame *P*(*FRF*) = 0.204, was determined as the frequency of FRFs in 5,550 basecalls (10 most likely basecalls for 555 multiple flowgram alignments). Ultimately this gave *P*(*CBS*|*FRF*) = 0.489, and in a similar way the likelihood of a sequence being correctly basecalled if no full-length ORF was present, was calculated to *P*(*CBS*|*noFRF*) = 2.58e-4.

#### Calculating sequence likelihoods using HMM match as feature

Checking for the presence of an open reading frame is one way of ensuring that the called base sequence conforms to expectations based on prior knowledge (in this case, the very minimal requirement that the sequence can most probably be translated). Another way of using prior knowledge is to check how well the called base sequence matches the family of sequences of interest (in this case malaria DBLα-tags). To achieve this, we trained an HMM on a set of DBLα-tags, and then determined the relationship between possible HMM scores, and the probability that a sequence with this score is correctly called. HMMer v3.1 (Eddy, et al. 2013, http://hmmer.org/) was employed to train an HMM on 262 translated DBLα-tags from Asia, South America, as well as East and West Africa [13, 14]. This HMM was first used to score 6636 DBLα-tags from 227 genomes [12] and then the 10 most likely basecalls from 555 flowgram alignments. In both cases, the bit-scores were rounded to nearest integer, and set to zero if the sequence did not have a full-length open reading frame. All bit-scores *S*_*HMM*_ ≥170 were collected in one bin to assure, that unknown sequences scoring higher than any of the sequences used to make the score distributions, still benefit from the high score. A pseudocount of 1/10,000 was used for all integer scores, and for 0 < *S*_*HMM*_ < 170 the distributions were smoothed using a window size of 21 (Additional file 1: Figure S2). The likelihood of a sequence being correctly basecalled given any HMM score *S*_*HMM*_ (rounded to nearest integer) in the interval [0,170] was then calculated as:

8 $$ P\left(CBS\left|{S}_{HMM}\right.\right)=\frac{P\left({S}_{HMM}\left|CBS\right.\right)P(CBS)}{P\left({S}_{HMM}\right)} $$

where the likelihood of an HMM score given a true DBLα-tag *P*(*S*_*HMM*_|*CBS*) was determined from Additional file 1: Figure S2A, and the likelihood for encountering such a score in any of the ten most likely sequences *P*(*S*_*HMM*_) was determined from Additional file 1: Figure S2B. The prior likelihood of randomly picking the correct basecall was again *P*(*CBS*) ~ 1/10.

#### Post-processing of nucleotide sequences to remove PCR artifacts

For each isolate, the most likely correctly basecalled nucleotide sequences, found using equation 6, were first clustered by 96 % identity using Usearch v5.2.32 with seeds (cluster member with highest number of replicate reads) as output [15]. Then chimeras were removed as described below, and finally a coverage threshold of three reads per variant was used to remove the least supported sequences.

#### Removal of chimeras

Chimeras were removed using Uchime implemented in Usearch v5.2.32 [15, 16], first in *de-novo* mode where chimera detection is based on read abundance, all parents are expected to be present in the sequence set, and candidate parents must be at least 2x more abundant than the chimera candidate sequence. Subsequently, database mode was applied, where sequences are searched against self and chimeras are found irrespective of the abundance of the parents.

#### Predicting sequences with errors

Several prediction methods were tested to generate a sequence set with high confidence error-free sequences. The predictions were based on a set of sequence characteristics, including those provided by Multipass for each basecall alternative: *P*(*CBS*|*flows*), *P*(*CBS*|*feature*), number of sequences and maximal positional flow variance in the flowgram alignment, the ranking of the sequence according to flowgrams alone, and a Boolean value indicating if the sequence was unanimously selected as the most likely sequence with regard to both flows and features. From these sequence characteristics it was possible to distinguish sequences that were less likely to contain errors, and thus create a high confidence and quality subset of sequences.

Known control sample sequences were used in the training of classifiers implemented in mlpy v3.5.0 [17] to distinguish between sequences with and without error. The characteristics pertaining to each sequence were scaled to the interval [0, 1] prior to classification. To determine the optimal value for the diagonal linear discriminant analysis (DLDA) regularization parameter Δ [18], the logistic regression and support vector machine (SVM) cost of constraints violation parameter C, and the SVM radial basis function (RBF) kernel parameter γ, two rounds of 20 iterations 10-fold cross-validation were carried out with parameter value grid search in the range [2^-15^, 2^20^], first round using a coarse grid and next using a local fine grid around the initial optimum. The discriminant threshold (probability of belonging to either class) was set so all error-sequences in the training set were classified correctly, and the F_2_ measure was calculated to evaluate classifier performance. The F_β_ measure is the weighted harmonic mean of sensitivity and precision, with β = 2 giving more weight to sensitivity [19]. Thus, optimal parameter values were selected as the ones giving the highest mean F_2_ score over the last 20 iterations cross-validation. The optimal parameters were used for training during leave-one-out cross-validation, as well as for the final classifier training on all control sample sequences.

#### Assembly of genomic Illumina sequencing data

European Nucleotide Archive (ENA) accession numbers for 227 samples published in ([12] Additional file 1: Table S12) was used to download the raw Illumina read sequences from the published study. Samples containing multiple accession numbers, hence referring to several separate sequencing experiments, were merged into one read file dataset. A few ENA accession numbers did not contain any data and were disregarded. Using a de Bruijn graph-based *de-novo* assembler, Velvet (v1.2.07) [20], all samples were *de-novo* assembled. We did not perform any quality trimming prior to the *de-novo* assembly, as a small benchmark showed that it reduced the amount of genes of interest. For each dataset several assemblies were run using the procedure published in [21]. This implies that for each dataset velveth was executed using *k*-mer sizes in the range from 33 to 80 % of the average read length. Next, the velvetg step was run using the parameters: cov_cutoff = 5, exp_cov = auto and min_contig_lgth = 100. Based on the number of contigs, the best cumulative rank for N50 and the length of the largest contig, the final best assembly was selected.

## Results

### Combining Multipass with models for protein coding DNA

**Fig. 1:**
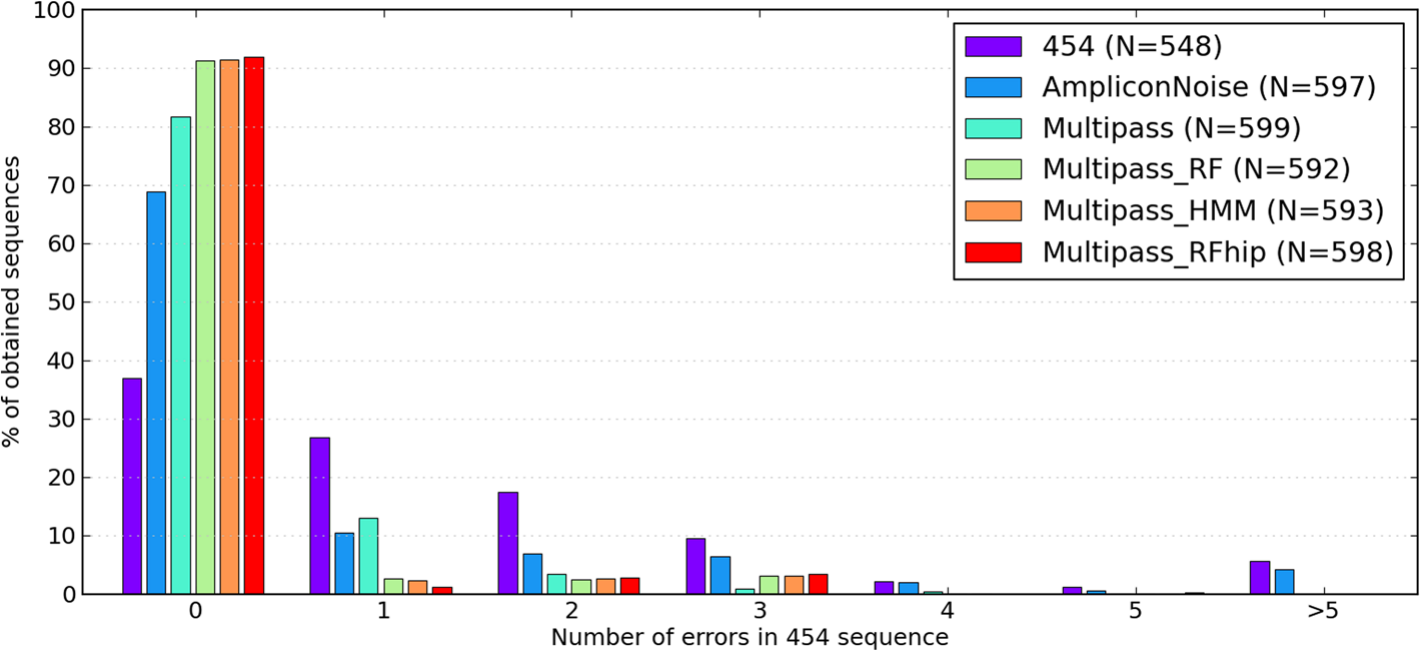
Accuracy of *Plasmodium falciparum* reference strain amplicon resequencing using different basecalling methods. Shown for each basecalling method is the fraction of all sequences (provided in the legend as N) with a given number of errors. See Additional file 1: Figure S3 with log-scale for detailed frequencies of sequences with multiple errors

Three *Plasmodium falciparum* laboratory reference strains were resequenced multiple times using bi-directional 454 FLX Titanium amplicon sequencing (12 samples containing one or mixtures of isolates 3D7, DD2 and HB3). Specifically, a ~370 nucleotide region (st.dev. 25 nt, min. 319 nt, max. 468 nt) in the hyper-variable *var* gene family was targeted, encoding the malaria antigen PfEMP1, of which each parasite has 50-60 variants. The flowgram data was demultiplexed, MID-tags and primer sequences were removed, and reverse flowgrams were reverse complemented as described in the Methods section. The resulting median sample coverage was 2956 reads (Additional file 1: Table S1). Flowgrams from each sample were then subjected to different basecalling methods as described below, and the resulting nucleotide sequences were subjected to denoising and chimera removal to eliminate PCR artifacts. This resulted in a total yield from the 12 samples of between 548 and 599 sequence variants, with varying degrees of accuracy compared to the known reference database sequences (Fig. 1).

Direct 454 basecalling of each read into nucleotides, by deriving homopolymer lengths from flow values rounded to nearest integer, and subsequent denoising, resulted in a set of *var* sequences where 37.0 % matched the known database sequences perfectly (Fig. 1) while sequences in average had 1.49 errors (st.dev. 1.99, max. 14) (Additional file 1: Figure S3). In this approach, reads originating from the same template sequence was determined only by clustering after flowgram basecalling, and this yielded 548 sequence variants.

The current state of the art method AmpliconNoise clusters and aligns the flowgrams before calculating the most likely nucleotide sequence from each alignment. Applying AmpliconNoise to the combined forward and reverse flowgrams nearly doubled the number of error-free sequences to 68.8 % (Fig. 1), and a mean error count of 0.935 (st.dev. 2.04, max. 13) was observed (Additional file 1: Figure S3). Flowgram clustering increased the yield to 597 variants, most likely because the more correct sequences gave larger clusters during denoising, leading to a higher number passing the cluster size threshold of 3 reads.

Multipass was then tested, using the initial flowgram clustering of AmpliconNoise, but with novel implementation of flowgram alignment and calculation of the *N* most likely nucleotide sequences from each alignment. Multipass uses updated probability distributions for flow signals originating from homopolymers with length >5 nucleotides (see Methods). Hence a considerable improvement in sequence accuracy was observed for this dataset by simply selecting the most likely nucleotide sequence given by Multipass, increasing the fraction of perfectly basecalled sequences to 81.8 % (Fig. 1) and reducing the mean number of errors per sequence to 0.258 (st.dev. 0.644, max. 5) (Additional file 1: Figure S3).

**Fig. 2:**
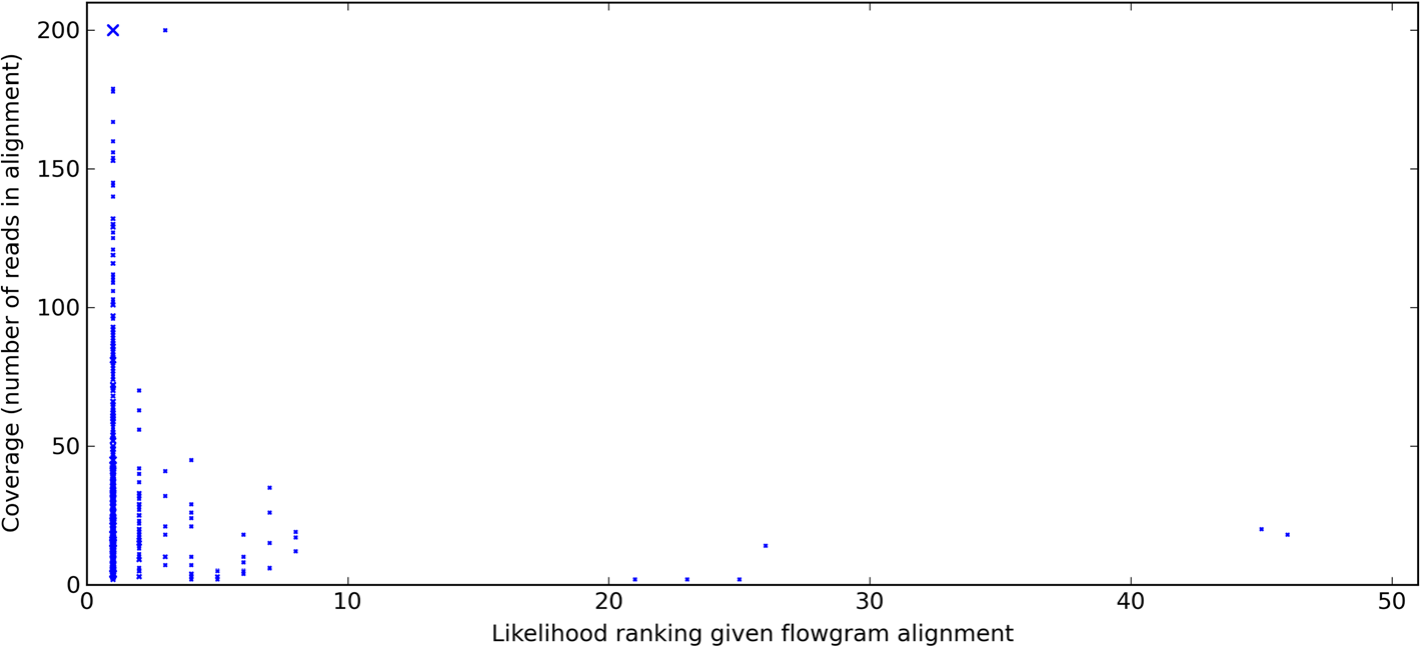
Ranking of the correct basecall according to *P*(*CBS*|*flows*). Upon flowgram clustering and alignment, Multipass was employed to calculate the fifty most likely basecalls given each flowgram alignment. The likelihood ranking of the correct basecall is shown against the number of flowgrams in the alignment. Maximally two hundred flowgrams from each cluster were used for alignment. The marker size is proportional to the abundance at a point

Using Multipass to calculate the most likely nucleotide sequences from each flowgram alignment revealed that, especially for low coverage sequences, a considerable fraction of correct basecalls were ranked not as the most likely, but in the top ten most likely sequences (Fig. 2). Therefore, Multipass was set to calculate the *N* = 10 most likely sequences, and in order to give the correctly basecalled sequences more support, expected sequence features were employed. So for each of the 10 most likely sequences, the likelihood of the sequence being correct given the flowgrams *P*(*CBS*|*flows*) was combined with the likelihood of the sequence being correct given a sequence feature *P*(*CBS*|*feature*), to give *P*(*CBS*|*flows*,*feature*) by which the most likely correct basecall was chosen. Any sequence feature enabling discrimination between correct and incorrect basecalls could be utilized.

The first feature used in the model was the presence or absence of a full-length open reading frame, where the probability of having a FRF in a correctly basecalled sequence *P*(*FRF*|*CBS*) was determined by the frequency of FRFs in ~6800 DBLα-tag sequences obtained by whole genome Illumina sequencing [12]. Out of the 10 sequences provided by Multipass, the one with highest likelihood given flowdata and presence of full-length open reading frame *P*(*CBS*|*flows*,*FRF*) was selected. This approach raised the basecalling accuracy to 91.2 % correct sequences (Fig. 1, RF), and a mean of 0.189 errors per sequence (st.dev. 0.671, max. 5). Since the Illumina sequences used to establish *P*(*CBS*|*FRF*) may contain errors disrupting the full length reading frame, the true *P*(*FRF*|*CBS*) could be higher than the one used above. Therefore we tried an arbitrary high *P*(*FRF*|*CBS*) = 1-1e-150, which gave an increase to 92.0 % correct sequences (Fig. 1, RFhip), however the mean number of errors per sequence also increased to 0.192 (st.dev. 0.700, max. 5), indicating that this model aggravated the condition of erroneous sequences (Additional file 1: Figure S3).

The second feature tried in the model was the match to a profile hidden Markov model (HMM) of the expected amino acid sequence, generated from DBLα-tags obtained from a small global selection of field isolates. This feature should be even more sensitive to frameshifts than FRF, since the HMM score is lowered even if the full-length open reading frame is retained upon introduction of an indel. The likelihood of a sequence being correctly basecalled given an HMM match score of a certain magnitude *P*(*CBS*|*S*_*HMM*_) was determined from score distributions (Additional file 1: Figure S2) as described in the Methods section. By selecting the most likely sequence given flowgrams and HMM match *P*(*CBS*|*flows*,*S*_*HMM*_), the accuracy reached 91.4 % correct sequences (Fig. 1), and a mean of 0.189 errors per sequence (st.dev. 0.673, max. 5).

### Generation of high confidence error-free sequence subset

In some cases it may be desirable to only work with sequences that are virtually free of errors, for example to enable translation of DNA sequences with low risk of obtaining amino acid sequence from the wrong reading frame. Multipass provides a list of characteristics for each of the most likely basecalled sequences, such as *P*(*CBS*|*flows*) and maximal positional flow variance in the flowgram alignment. These characteristics are good sequence quality indicators, and they were used together with sequence feature characteristics to train a predictor to distinguish sequences with low likelihood of error, and thus create a high confidence and quality subset of sequences.

**Fig. 3:**
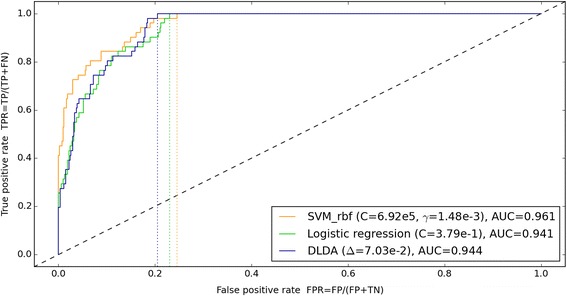
Receiver operator characteristics for prediction of incorrect sequences. Performance of three machine learning methods applied to differentiate between sequences with and without error in *var* sequences generated using Multipass basecalling. The classifiers were trained on a number of characteristics provided for each sequence, such as read coverage and maximal positional flow variance. Positive (P) and negative (N) refers to sequences with and without error, respectively. True (T) and false (F) refers to correct and incorrect predictions, respectively. For each method, the lowest false positive rate with perfect classification of the erroneous sequences is indicated (dotted lines)

A set of 592 known control sample sequences, of which 51 contained errors, was used to train various predictors to distinguish between sequences with and without error. Optimal training parameter values were found for each method by parameter grid search and 10-fold cross-validation. Leave-one-out cross-validation with the found optimal parameters resulted for diagonal linear discriminant analysis (DLDA) in a single misclassification of an error sequence (sensitivity = 98.0 %) and a specificity of 79.5 %, thus scoring higher than logistic regression (sensitivity = 98.0 %, specificity = 76.7 %) and a kernel support vector machine (sensitivity = 94.1 %, specificity = 75.4 %). Finally, a DLDA model trained on all control sample sequences and tested on the training sequences, gave a sensitivity of 100 % with specificity 79.5 %, compared to a specificity of 76.9 % for logistic regression and 75.4 % for the kernel support vector machine (Fig. 3). Thus, the DLDA classifier was found to generate a slightly larger error-free sequence set than the other two classifiers. Using the DLDA model on the sequence characteristics, a high confidence error-free subset of sequences could be delineated, missing only 20 % of the correct sequences.

## Discussion

As our knowledge about genomics and genetics increases we can more confidently predict what occurs in those realms, and when prior knowledge becomes so substantial that our expectations can outweigh the uncertainties of novel raw sequencing data, it makes sense to take advantage of both types of information in the interpretation of such new data.

Prior knowledge about the sequencing target region has not previously been employed in the basecalling process, in some cases due to the limited availability of such knowledge, though most likely also because it requires custom treatment of each individual sequencing project and no tools have been available for this purpose.

Basecalling uncertainties have hitherto mainly been given as positional quality scores, however such notation does normally not provide information about the nature (insertion, deletion, or nucleotide type) and likelihood of the alternative sequences, so this format omits information potentially important in downstream modelling.

Multipass generates the most likely sequences given the sequencing data in a fully probabilistic fashion, retaining information about both type and quantity of the sequence data uncertainty for downstream modelling and hypothesis testing. Each of the ten most likely sequences are hypotheses about the identity of the original template sequence in the sample, and we estimate the support for each hypothesis taking both sequencing signals and prior knowledge into account.

The *Plasmodium falciparum* genome contains a high concentration of long homopolymers [22], and even though the concentration is lower in coding DNA, it is still high compared to other common genomes (Additional file 1: Figure S4). Long homopolymer tracts are prone to indels, most likely due to polymerase slippage [23], and the high prevalence of these regions in *var* genes could potentially be a mechanism by which parasites generate antigenic diversity. However, disruption of the full-length open reading frame was rarely found in six thousand DBLα-tags from 227 field isolates. In yeast wildtype cells with functional DNA mismatch repair machinery, indels causing frameshift are efficiently repaired [24, 25]. It seems likely that a similar mechanism exists in *Pf*, which maintains a full-length ORF in more than 99.9 % of DBLα-tags.

The reduced intensity and high variance signal we obtained from long homopolymers may in part be caused by polymerase slippage during target PCR amplification and emulsion PCR (where no DNA mismatch repair machinery is present), possibly in combination with other factors such as incomplete incorporation of nucleotides during the flow (in marked cases visible in flowgrams as splitting of flows into multiple cycles, which was observed more frequently for long homopolymers).

Multipass was developed for Roche 454 pyrosequencing, and can with minor alterations be adapted to the similar IonTorrent flowgram data. The concept of calculating the most likely basecalls and their probability could also be implemented for other unrelated sequencing technologies, which would enable more optimal handling of sequencing uncertainties in downstream modeling. It would also be advantageous to use prior knowledge about the target region to assist basecalling in other sequencing technologies. Any sequencing project where excessive prior knowledge is available can potentially benefit from this approach in the form of higher sequence accuracy. One particularly exciting application of amplicon sequencing in a setting with extensive prior knowledge is the deep sequencing of human immune repertoires. In such a project, Multipass could be employed to provide detailed information about sequence uncertainties in a probabilistic model of VDJ recombination, to explore how germline gene repertoires are associated with immune target specificity.

## Conclusions

Here we show that Multipass can make more accurate basecalls for amplicon pyrosequencing data using updated flow signal distributions. In addition, it is demonstrated how this probabilistic framework facilitates downstream modeling that takes sequence uncertainties into account, and how a model can be built that further improves sequence accuracy using prior knowledge about the target region. Hopefully the methods described in this paper will be used to improve basecalling accuracy, and inspire new ways to incorporate sequencing uncertainties in modeling and hypothesis testing, both in the analysis of future sequencing data, as well as in reanalysis of the substantial amount of existing data.

## Availability of data and material

The datasets supporting the conclusions of this article are available in the NCBI Sequence Read Archive, [https://www.ncbi.nlm.nih.gov/bioproject/prjna317012].

## Additional files

Additional file 1: Supplementary data. This file contains supplementary Table S1 and Figures S1-S4. (PDF 781 kb) Additional file 2: Supplementary Figure S1A details. This file contains all details of Figure S1A. (PDF 1137 kb)

## Abbreviations

CBS: correctly basecalled sequence
DBL: Duffy binding-like
DLDA: diagonal linear discriminant analysis
ENA: European nucleotide archive
FRF: full-length open reading frame
HMM: hidden Markov model
HP: homopolymer
MID: multiplex identifier
ORF: open reading frame
Pf: *Plasmodium falciparum*
PTP: picotiter plate
RBF: radial basis function
SVM: support vector machine
