# Supplementary material for: Using expected sequence features to improve basecalling accuracy of amplicon pyrosequencing data
Source: BMC Bioinformatics. 2016 Apr 22;17:176. doi: 10.1186/s12859-016-1032-7 (PMC4841065; doi:10.1186/s12859-016-1032-7)
Supplement: Additional file 1: — Supplementary data. This file contains supplementary Table S1 and Figures S1-S4. (PDF 781 kb) [file 12859_2016_1032_MOESM1_ESM.pdf]

Using expected sequence features to improve basecalling accuracy of amplicon pyrosequencing data

Thomas S. Rask, Bent Petersen, Donald S. Chen, Karen P. Day, and Anders Gorm Pedersen

Supplementary data

Supp. Table S1. Resequenced *Plasmodium falciparum* samples

Sample #	Isolates[1]	Read coverage	MID.pool.plate	Left MID	Right MID	Accession[2]
1	3D7	2038	MID46-47.P1.apr13	TGACGTATGT	TGTGAGTAGT	SRR3317475
2	3D7	2028	MID54.P1.mar13	AGTGCTACGA	AGTGCTACGA	SRR3317479
3	3D7	2052	MID57.P1.apr11	CGCGTATACA	CGCGTATACA	SRR3317481
4	DD2	3387	MID47-47.P1.apr13	TGTGAGTAGT	TGTGAGTAGT	SRR3317472
5	DD2	1687	MID56.P3.mar13	CGCAGTACGA	CGCAGTACGA	SRR3317477
6	DD2	3500	MID57.P2.apr11	CGCGTATACA	CGCGTATACA	SRR3317486
7	HB3	5347	MID46-48.P2.apr13	TGACGTATGT	ACAGTATATA	SRR3317470
8	HB3	3002	MID55.P2.mar13	CGATCGTATA	CGATCGTATA	SRR3317478
9	HB3	15166	MID57.P3.apr11	CGCGTATACA	CGCGTATACA	SRR3322371
10	3D7+DD2	3923	MID57.P4.apr11	CGCGTATACA	CGCGTATACA	SRR3317485
11	DD2+HB3	2910	MID47-48.P2.apr13	TGTGAGTAGT	ACAGTATATA	SRR3317471
12	DD2+HB3	2264	MID57.P4.mar13	CGCGTATACA	CGCGTATACA	SRR3317484

[1]Samples were composed of one or two *Plasmodium falciparum* laboratory reference isolates.
[2]Sequences were deposited in the NCBI Sequence Read Archive: www.ncbi.nlm.nih.gov/sra

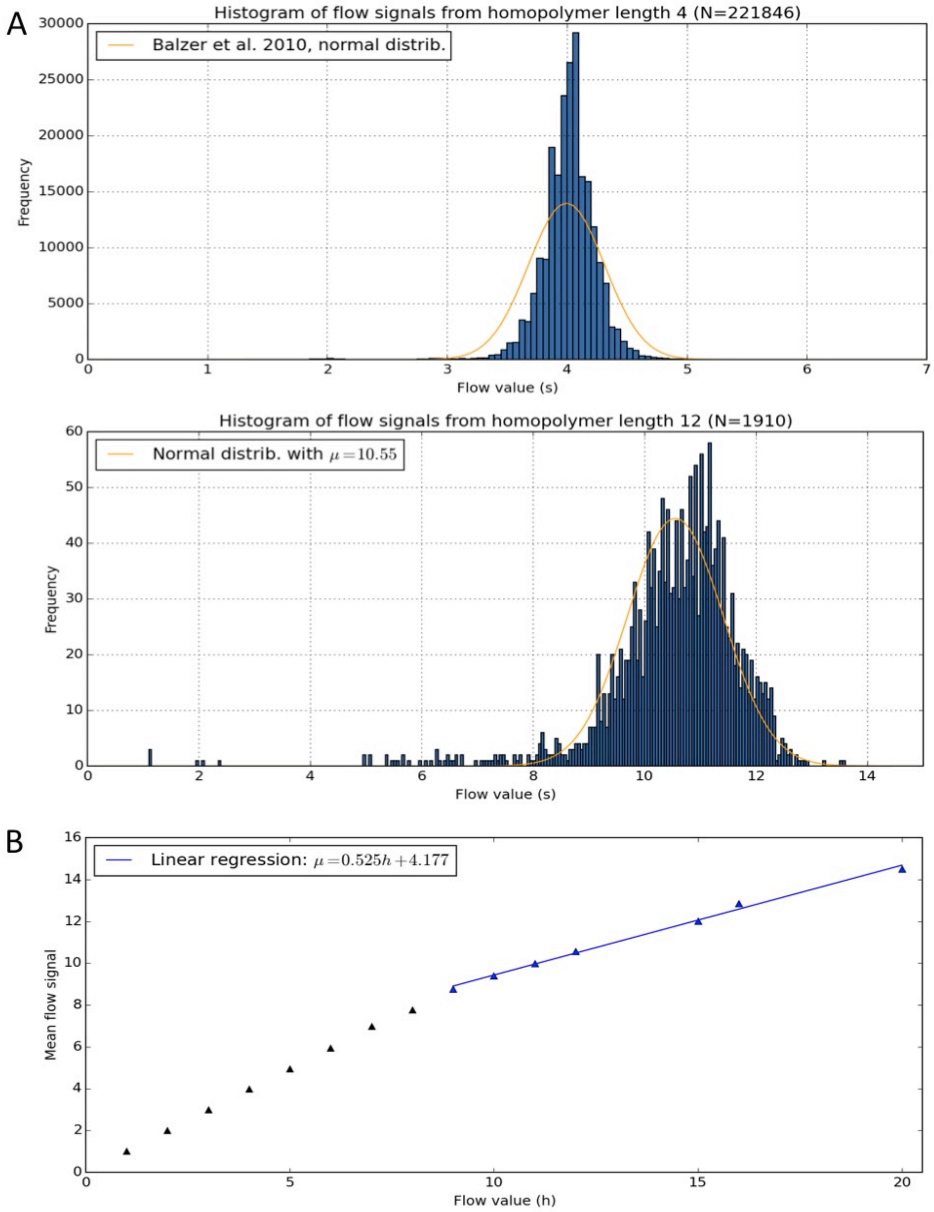

Supp. Fig. S1. (**A)** Flow signal histograms for homopolymers present in *var* reference sequences (h=4 and h=12 shown, see separate file for remaining homopolymer lengths). Normal and log-normal distributions as suggested by (Balzer, *et al.*, 2010) are shown for homopolymer length h≤5. For h>5, normal distribution means derived from the data were used with linearly extrapolated variance as suggested in (Balzer, *et al.*, 2010). (**B**) The normal distribution means for h≥9 could be described as a linear function of h with a lower inclination than that of h≤9.

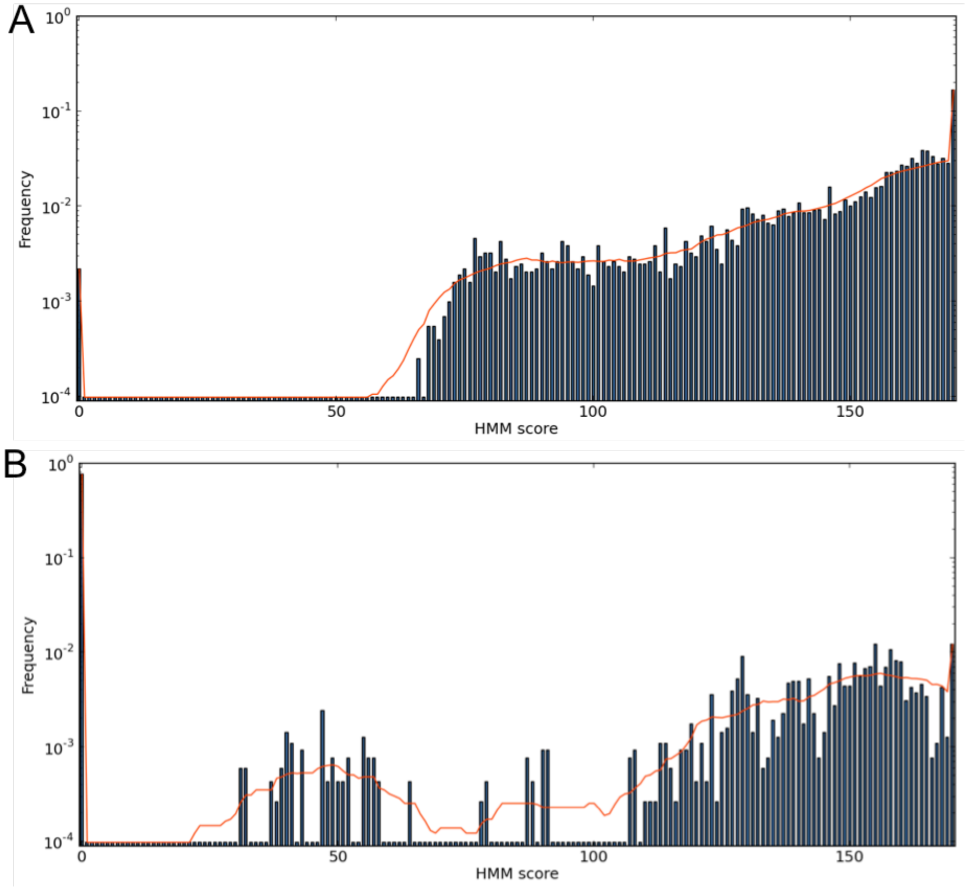

Supp. Fig. S2. HMM log-odds score histograms. The DBLα-tag HMM was matched against (**A**) 6636 DBLα-tags obtained by Illumina whole genome sequencing of 227 field isolates (Manske, *et al.*, 2012), and (**B**) Ten most likely basecalls for 555 flowgram alignments. Orange lines indicate the score frequencies used for calculation of $P(CBS|S_{HMM})$, where scores in the interval $0<S_{HMM}<170$ were subjected to smoothing.

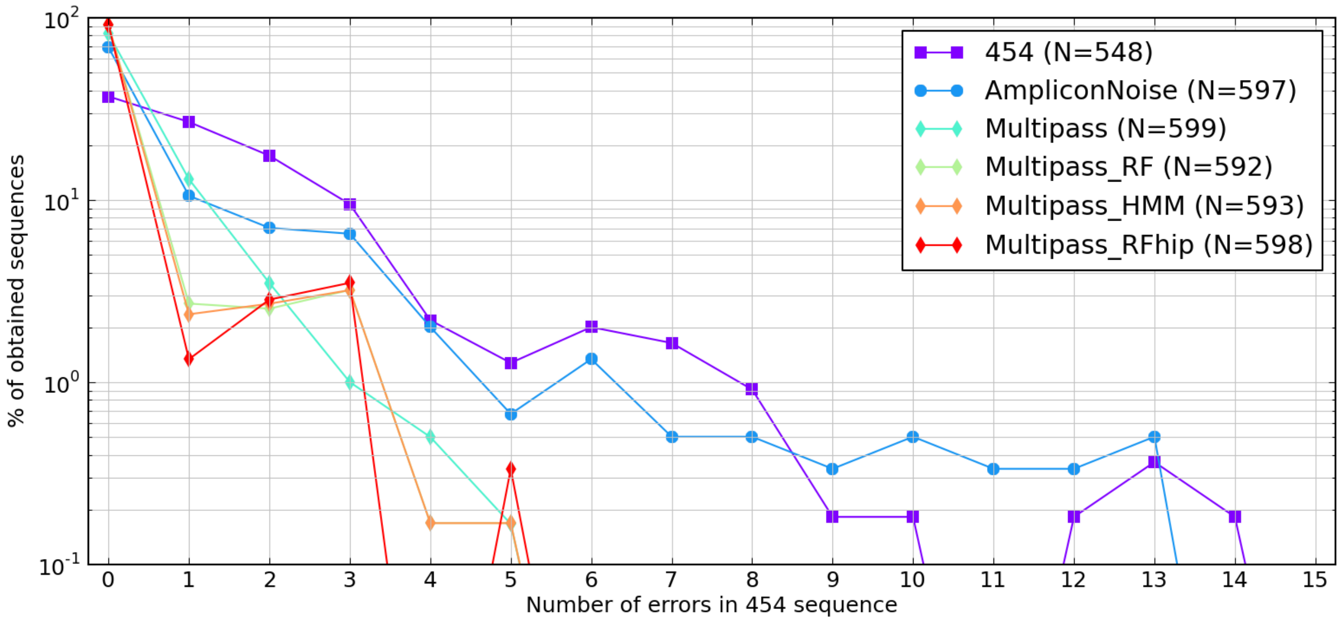

Supp. Fig. S3. Accuracy of *Plasmodium falciparum* reference strain amplicon resequencing using different basecalling methods. Based on same data as Figure 1, displayed with log-scale to give more detail regarding sequences with multiple errors.

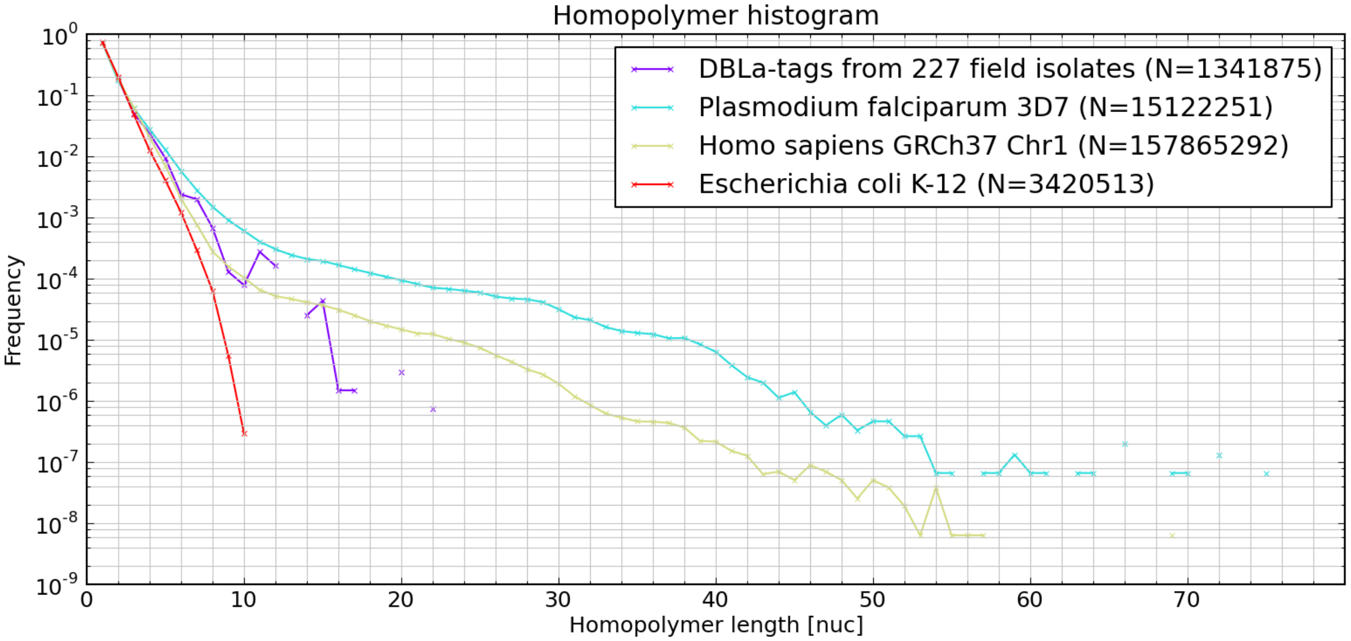

Supp. Fig. S4. Frequencies of different length homopolymers in the malaria parasite as well as human and bacterial genomes. The total number of homopolymers in each dataset is given as N in the legend.
