# Supplementary material for: Using expected sequence features to improve basecalling accuracy of amplicon pyrosequencing data
Source: BMC Bioinformatics. 2016 Apr 22;17:176. doi: 10.1186/s12859-016-1032-7 (PMC4841065; doi:10.1186/s12859-016-1032-7)
Supplement: Additional file 2: — Supplementary Figure S1A details. This file contains all details of Figure S1A. (PDF 1137 kb) [file 12859_2016_1032_MOESM2_ESM.pdf]

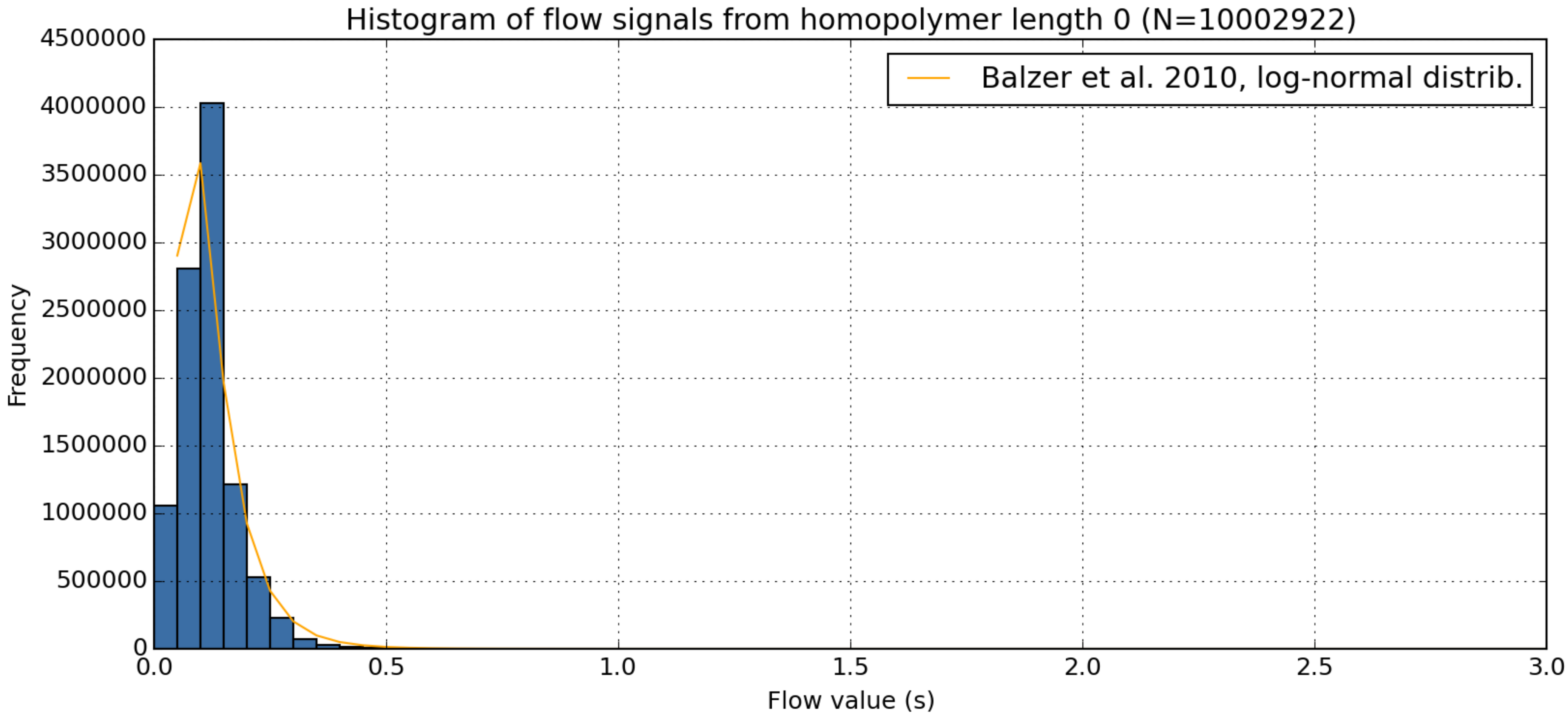
Histogram of flow signals from homopolymer length 0 (N=10002922)
Balzer et al. 2010, log-normal distrib.
Frequency
Flow value (s)
0
500000
1000000
1500000
2000000
2500000
3000000
3500000
4000000
4500000
0.0
0.5
1.0
1.5
2.0
2.5
3.0

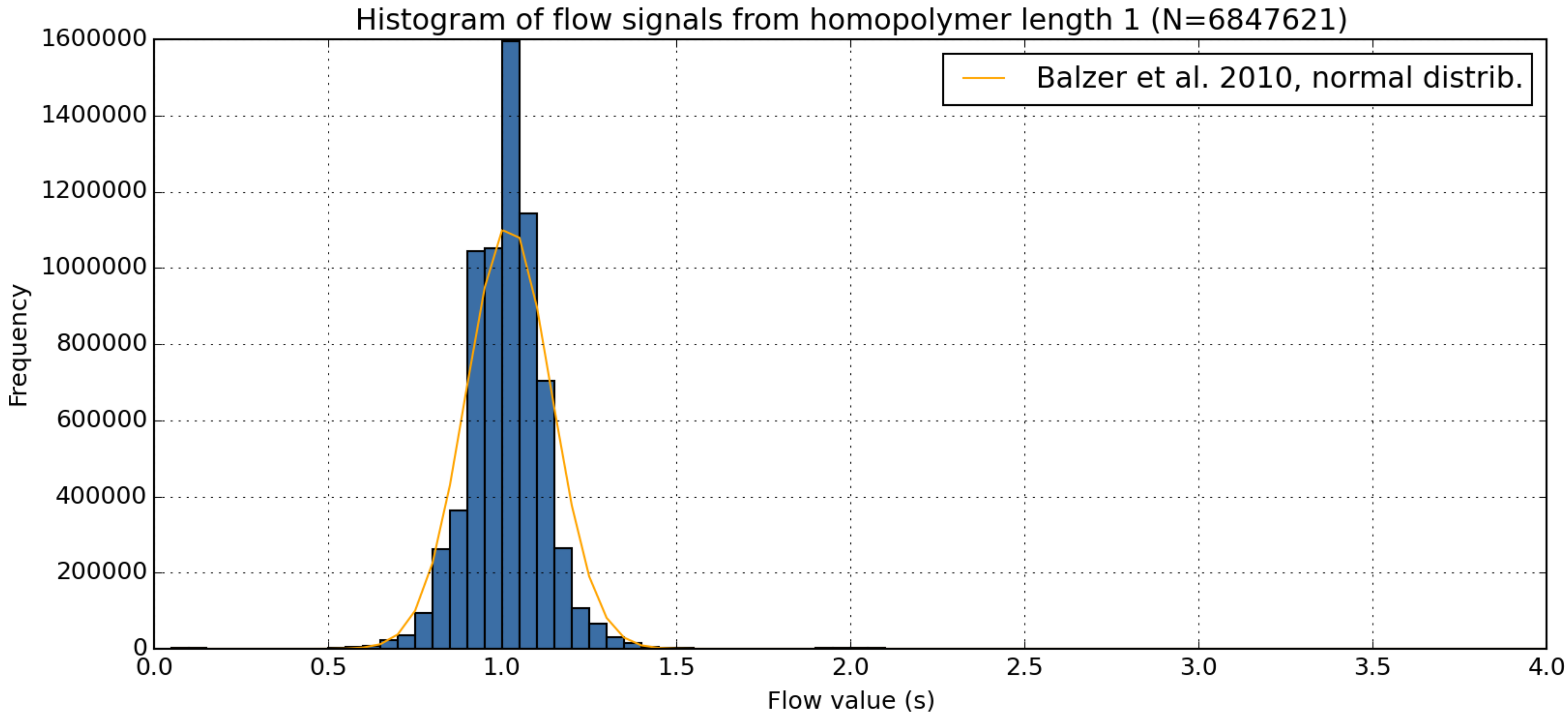
Histogram of flow signals from homopolymer length 1 (N=6847621)
Balzer et al. 2010, normal distrib.
Frequency
Flow value (s)
0
200000
400000
600000
800000
1000000
1200000
1400000
1600000
0.0
0.5
1.0
1.5
2.0
2.5
3.0
3.5
4.0

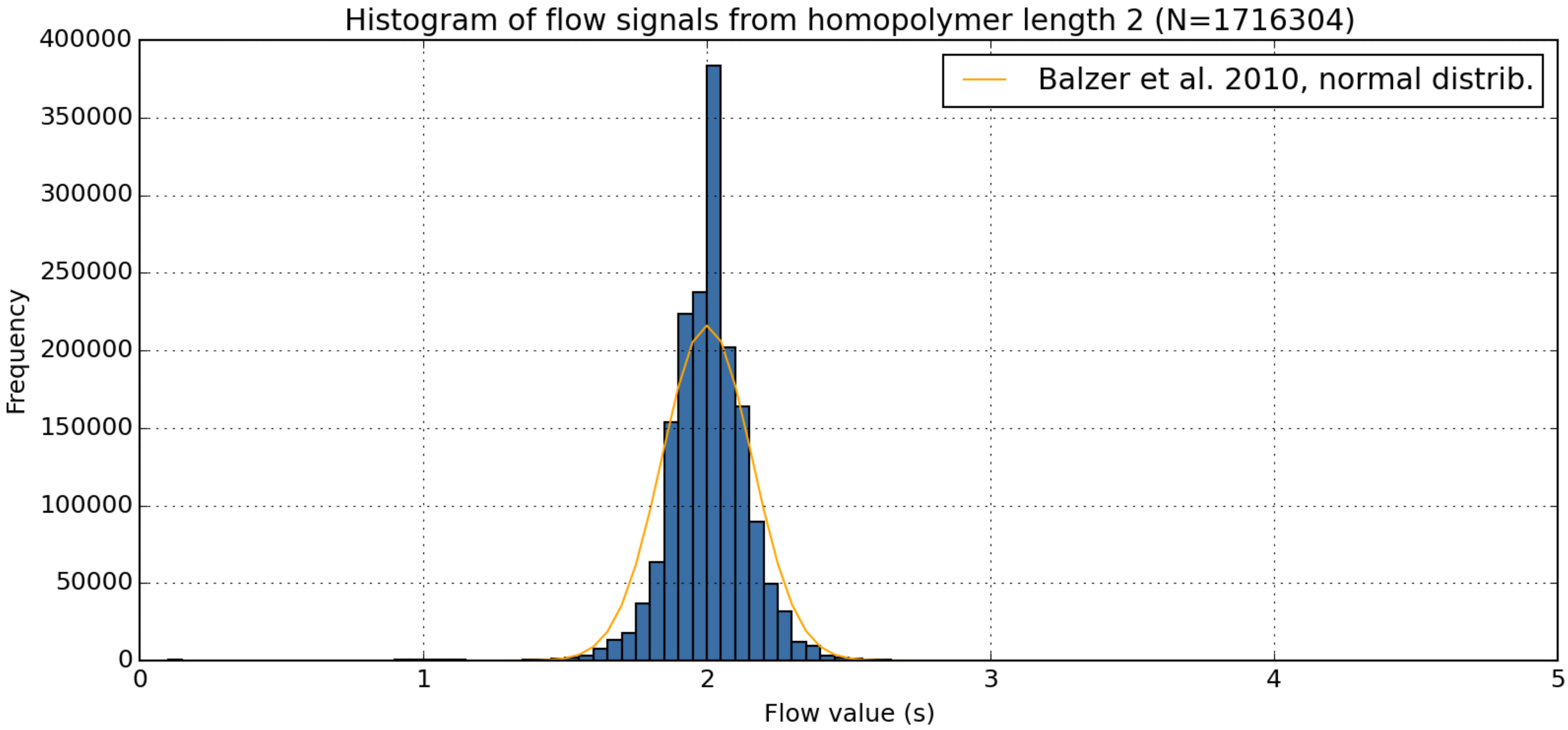
Histogram of flow signals from homopolymer length 2 (N=1716304)
Balzer et al. 2010, normal distrib.
Frequency
Flow value (s)
0
50000
100000
150000
200000
250000
300000
350000
400000
0
1
2
3
4
5

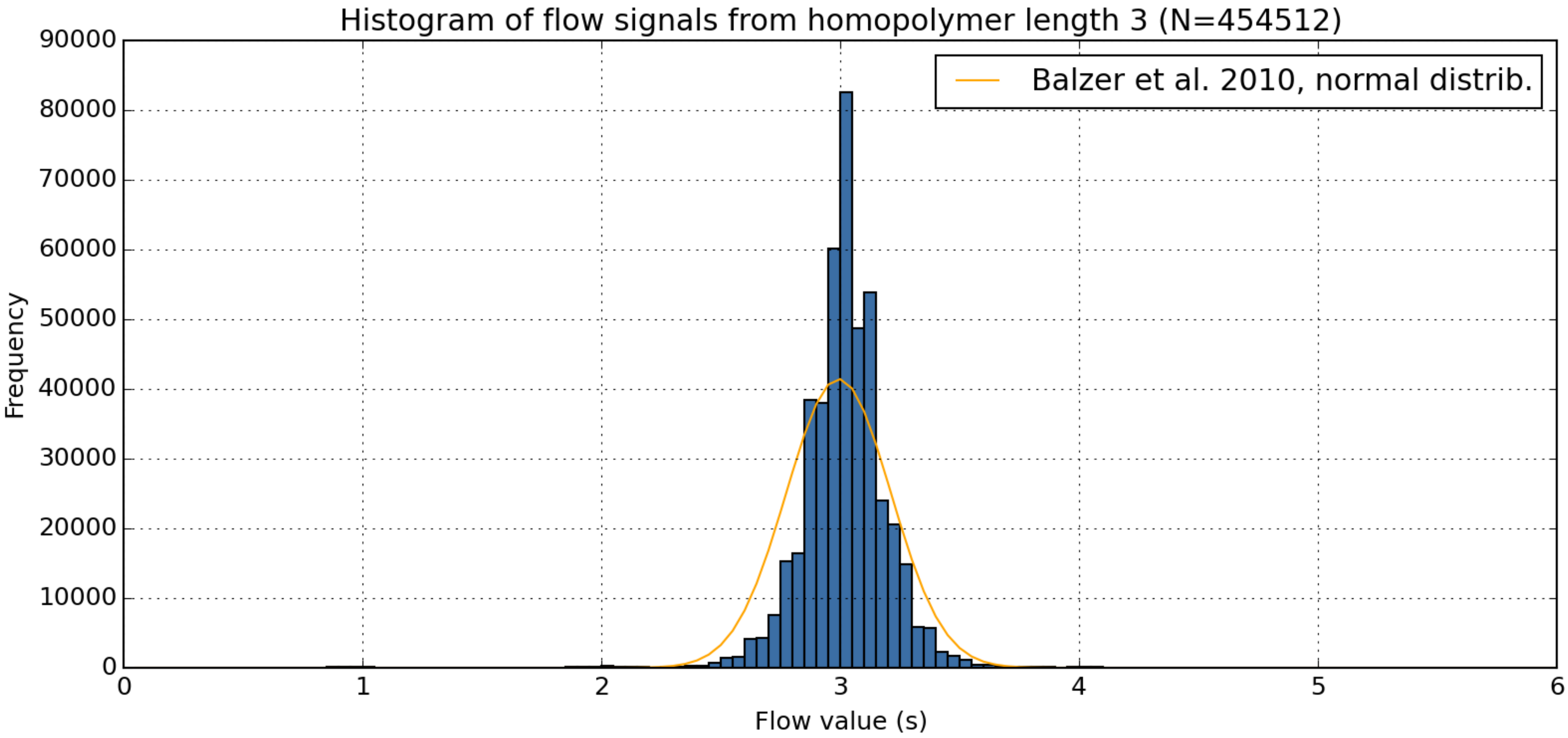

Histogram of flow signals from homopolymer length 3 (N=454512)
Balzer et al. 2010, normal distrib.
Frequency
90000
80000
70000
60000
50000
40000
30000
20000
10000
0
0
1
2
3
4
5
6
Flow value (s)

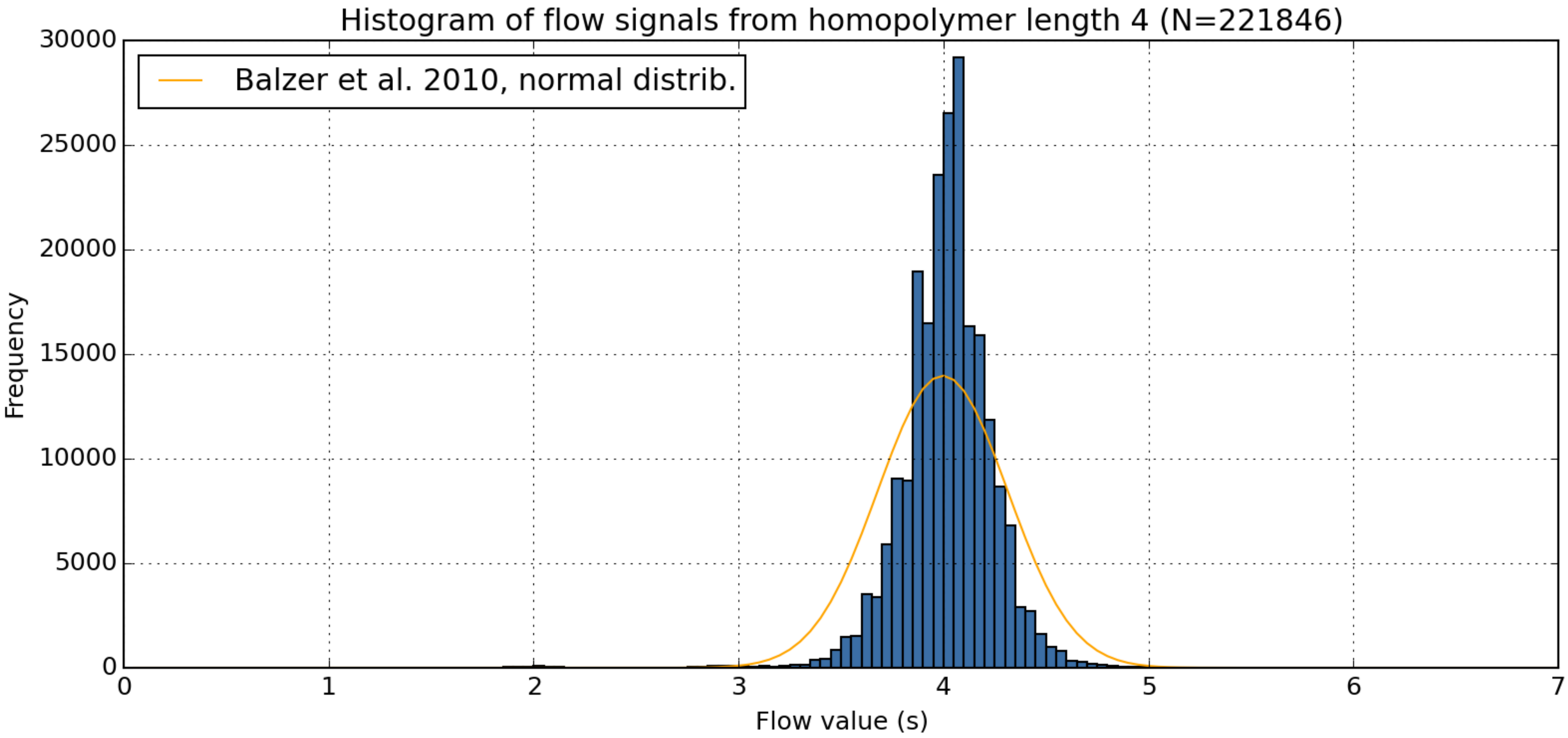

Histogram of flow signals from homopolymer length 4 (N=221846)
Balzer et al. 2010, normal distrib.
Frequency
30000
25000
20000
15000
10000
5000
0
0
1
2
3
4
5
6
7
Flow value (s)

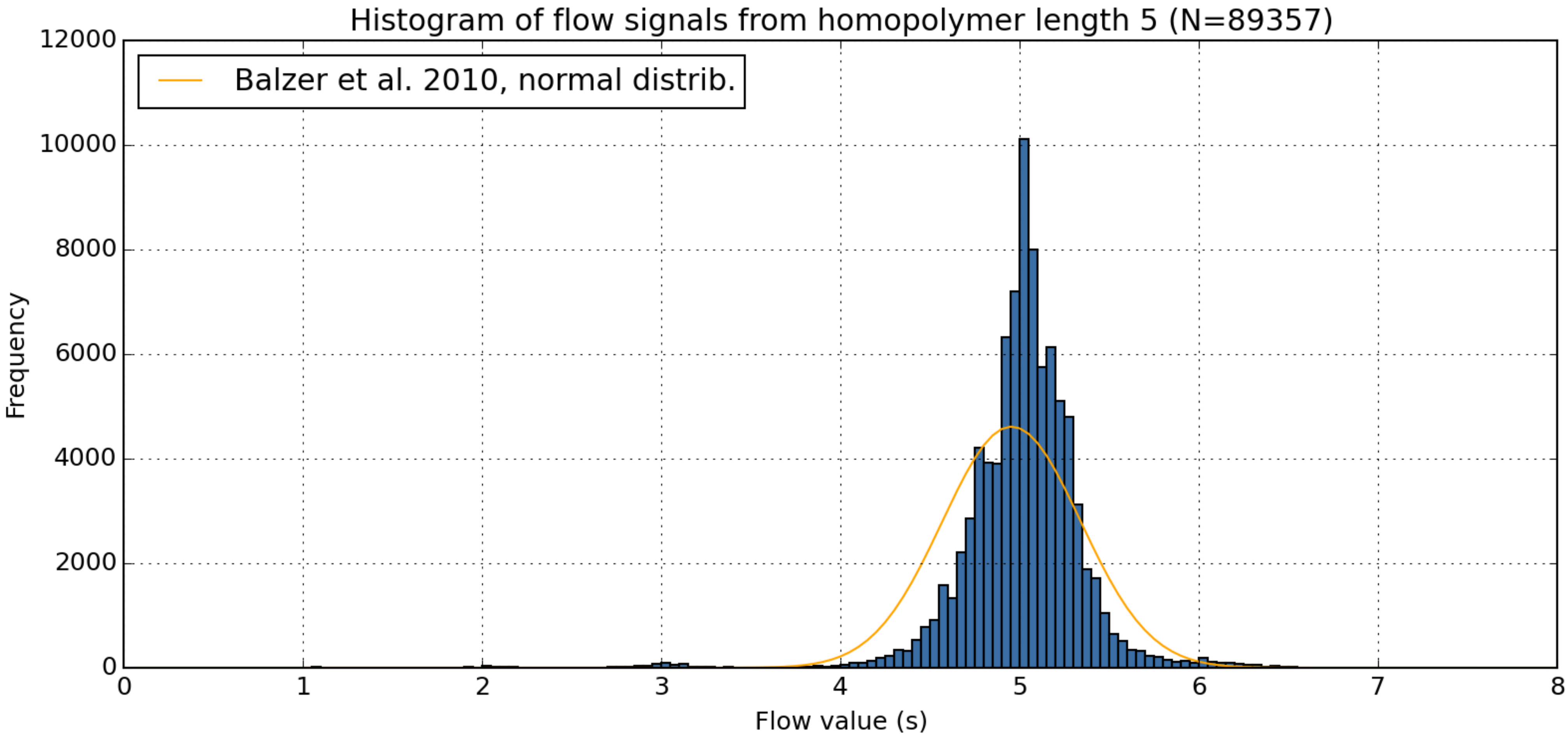

Histogram of flow signals from homopolymer length 5 (N=89357)
Balzer et al. 2010, normal distrib.
Frequency
Flow value (s)
0
2000
4000
6000
8000
10000
12000
0
1
2
3
4
5
6
7
8

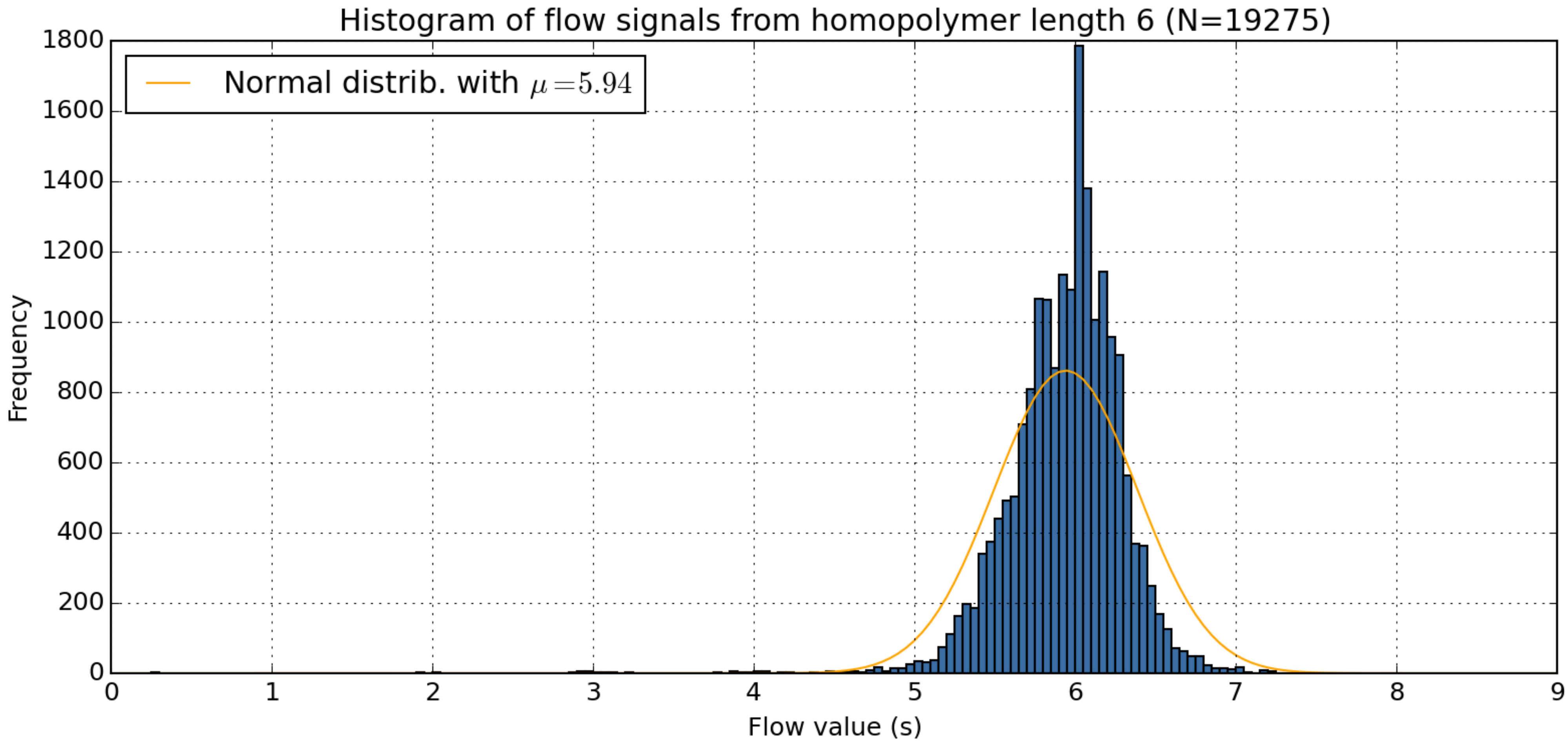

Histogram of flow signals from homopolymer length 6 (N=19275)
Normal distrib. with $\mu = 5.94$
Frequency
Flow value (s)
0
200
400
600
800
1000
1200
1400
1600
1800
0
1
2
3
4
5
6
7
8
9

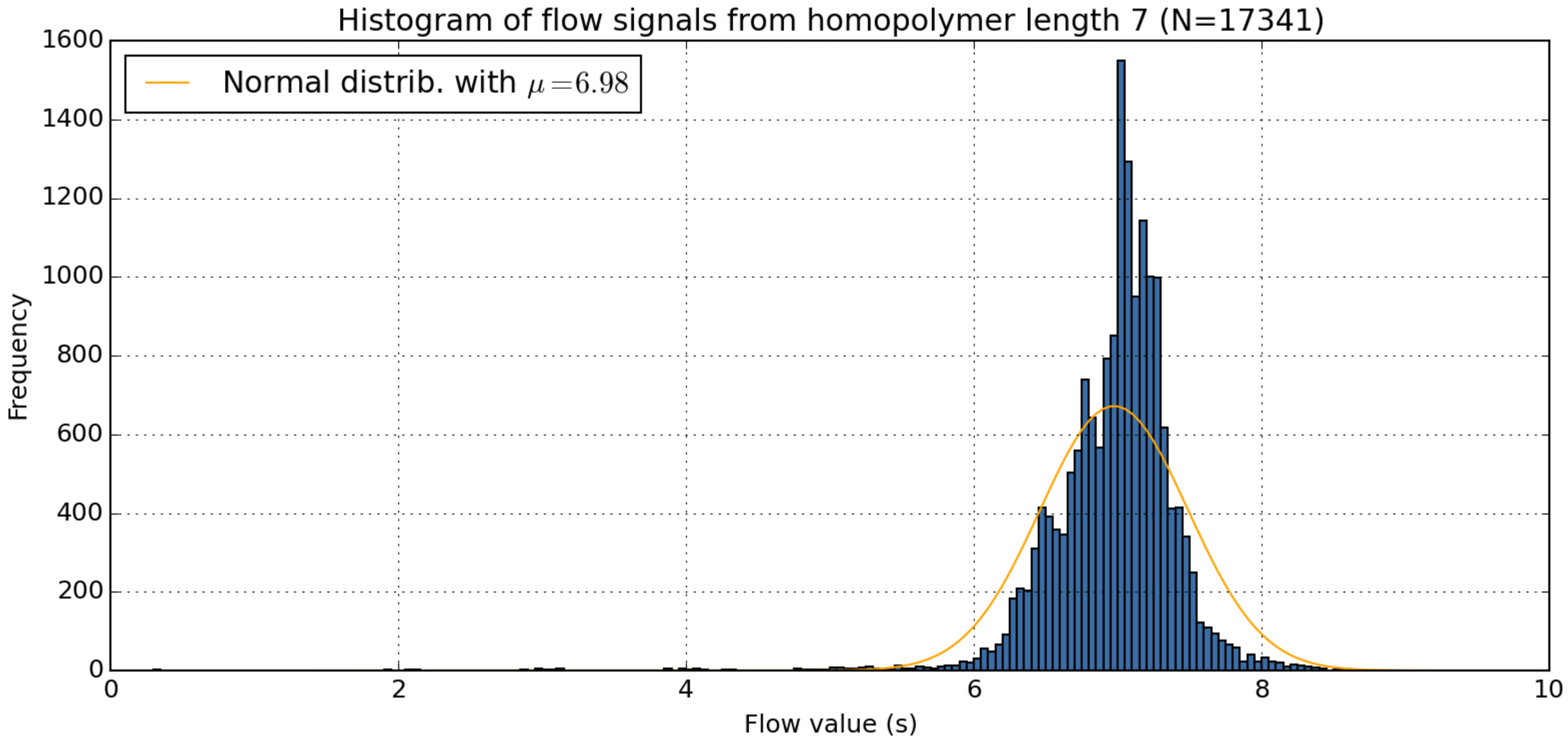

Histogram of flow signals from homopolymer length 7 (N=17341)
Normal distrib. with $\mu = 6.98$
Frequency
Flow value (s)
0
2
4
6
8
10
0
200
400
600
800
1000
1200
1400
1600

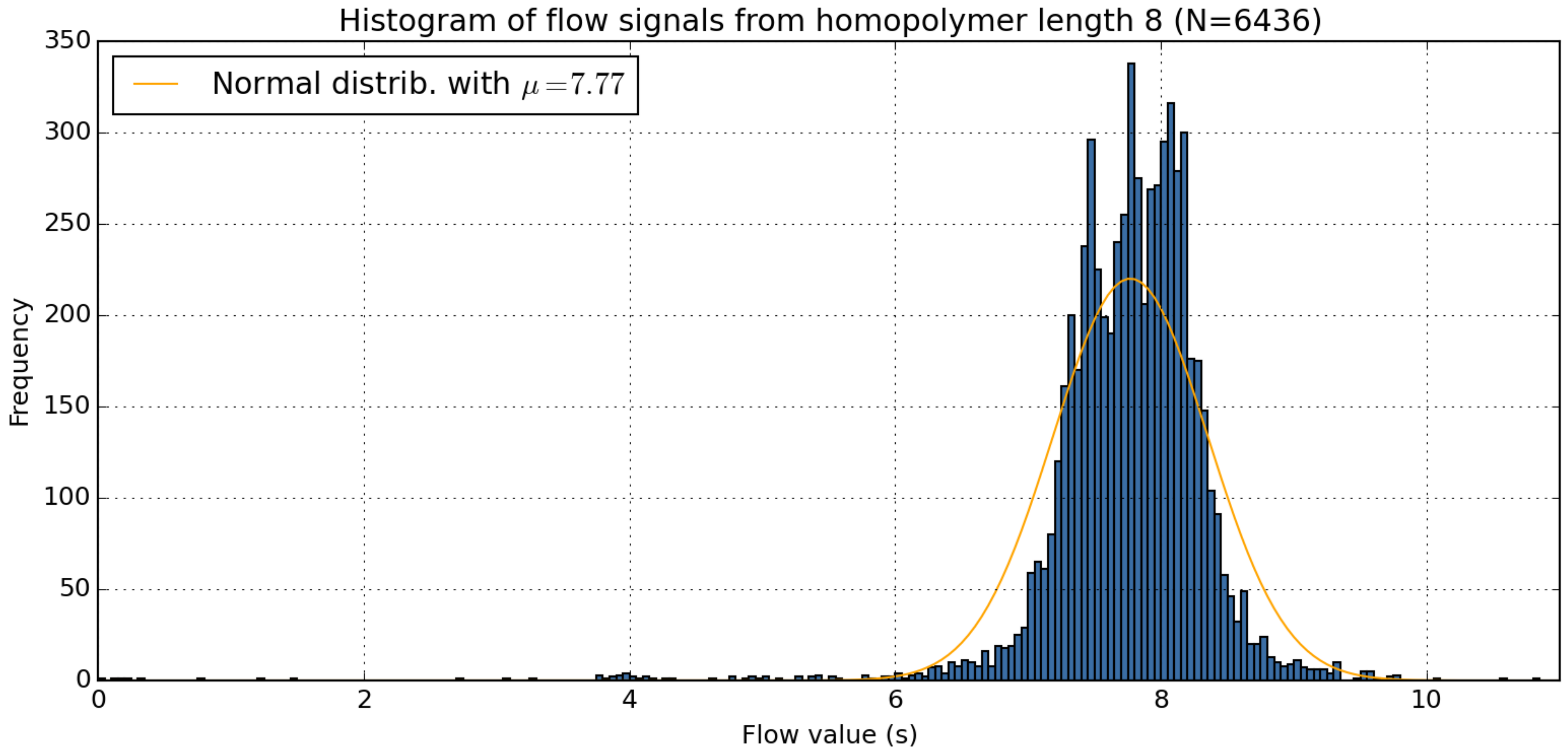
Histogram of flow signals from homopolymer length 8 (N=6436)
Normal distrib. with $\mu = 7.77$
Frequency
Flow value (s)
0
2
4
6
8
10
0
50
100
150
200
250
300
350

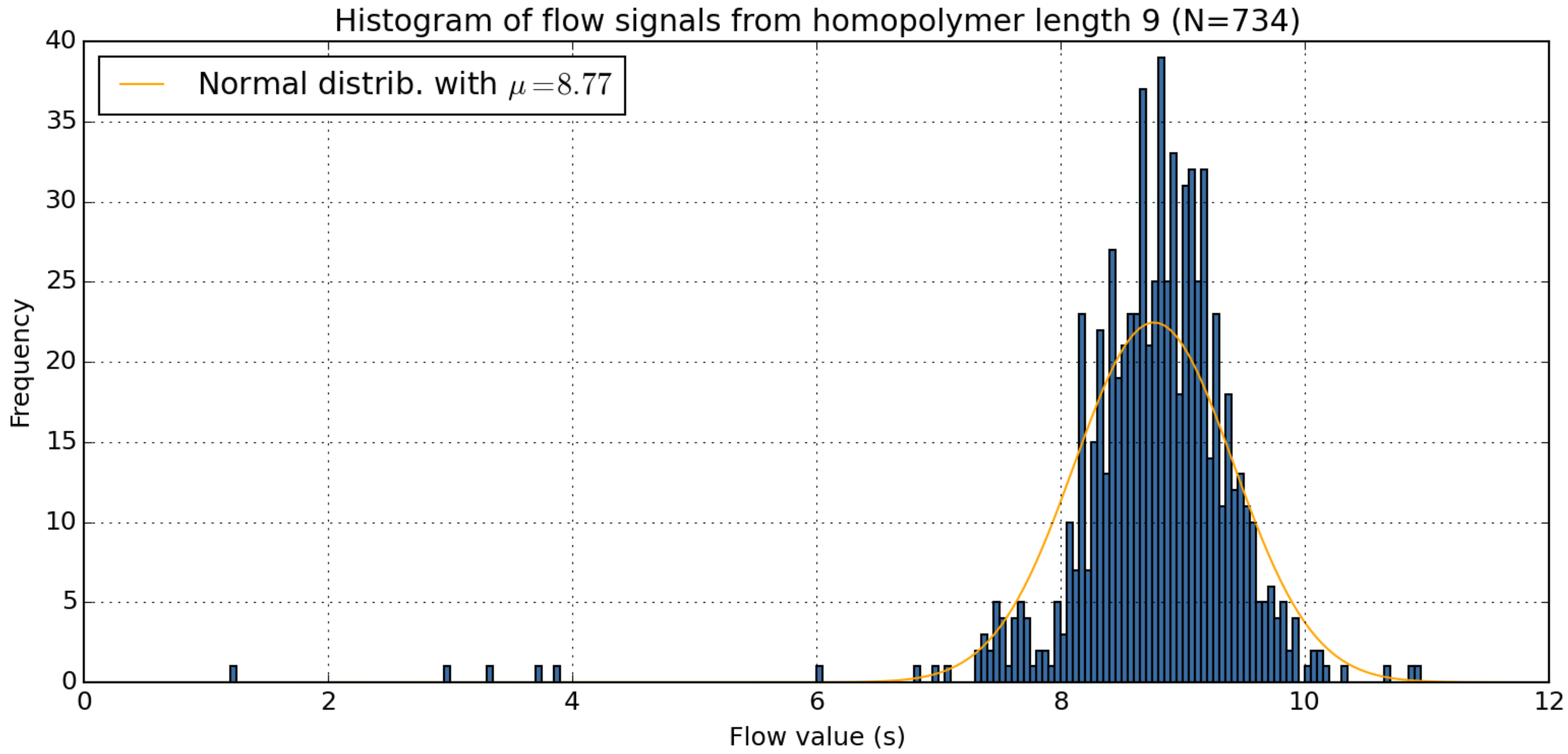

Histogram of flow signals from homopolymer length 9 (N=734)
Normal distrib. with $\mu = 8.77$
Frequency
Flow value (s)
0
5
10
15
20
25
30
35
40
0
2
4
6
8
10
12

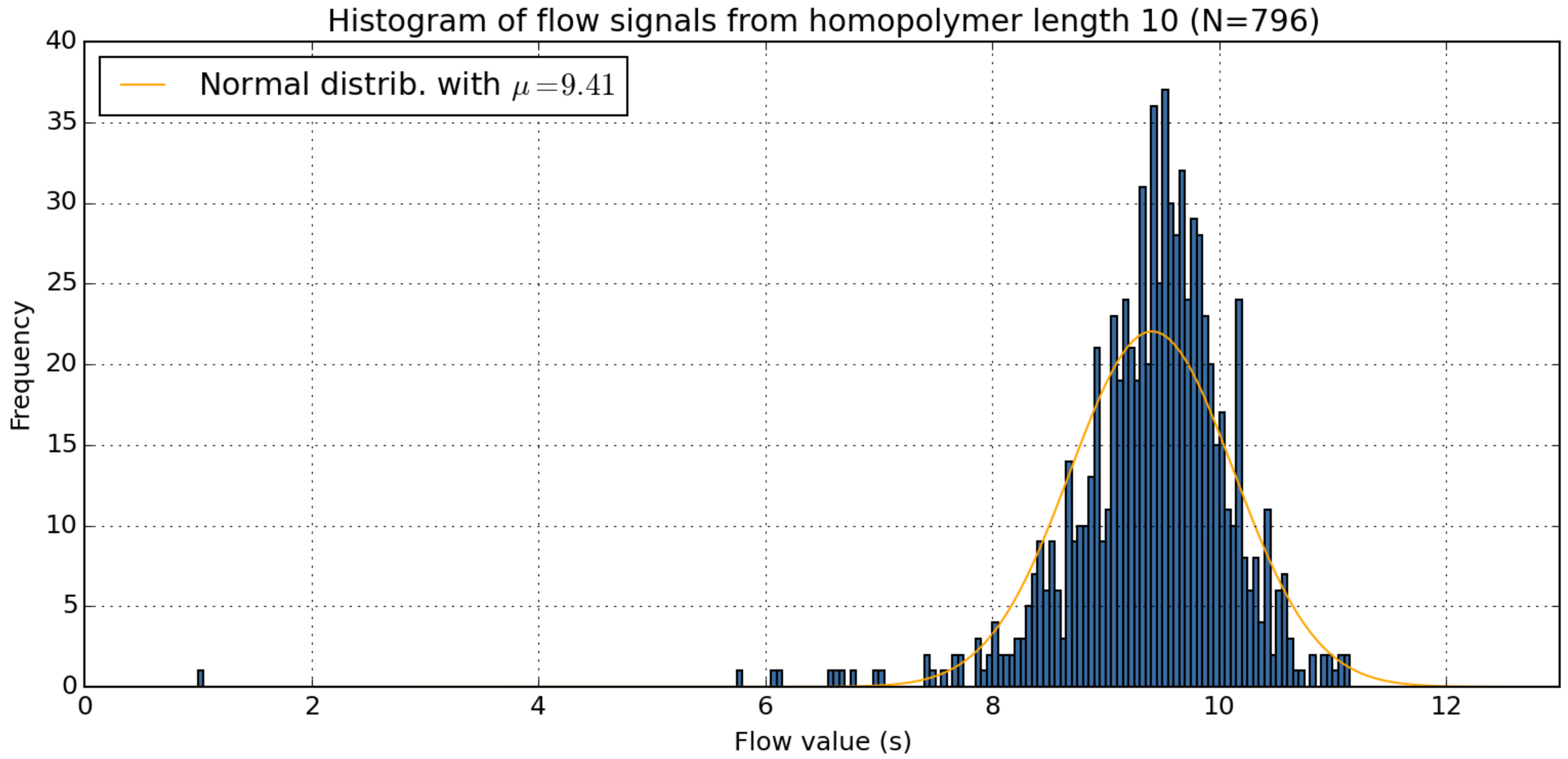
Histogram of flow signals from homopolymer length 10 (N=796)
Normal distrib. with $\mu = 9.41$
Frequency
Flow value (s)
0
2
4
6
8
10
12
5
15
20
25
30
35
40

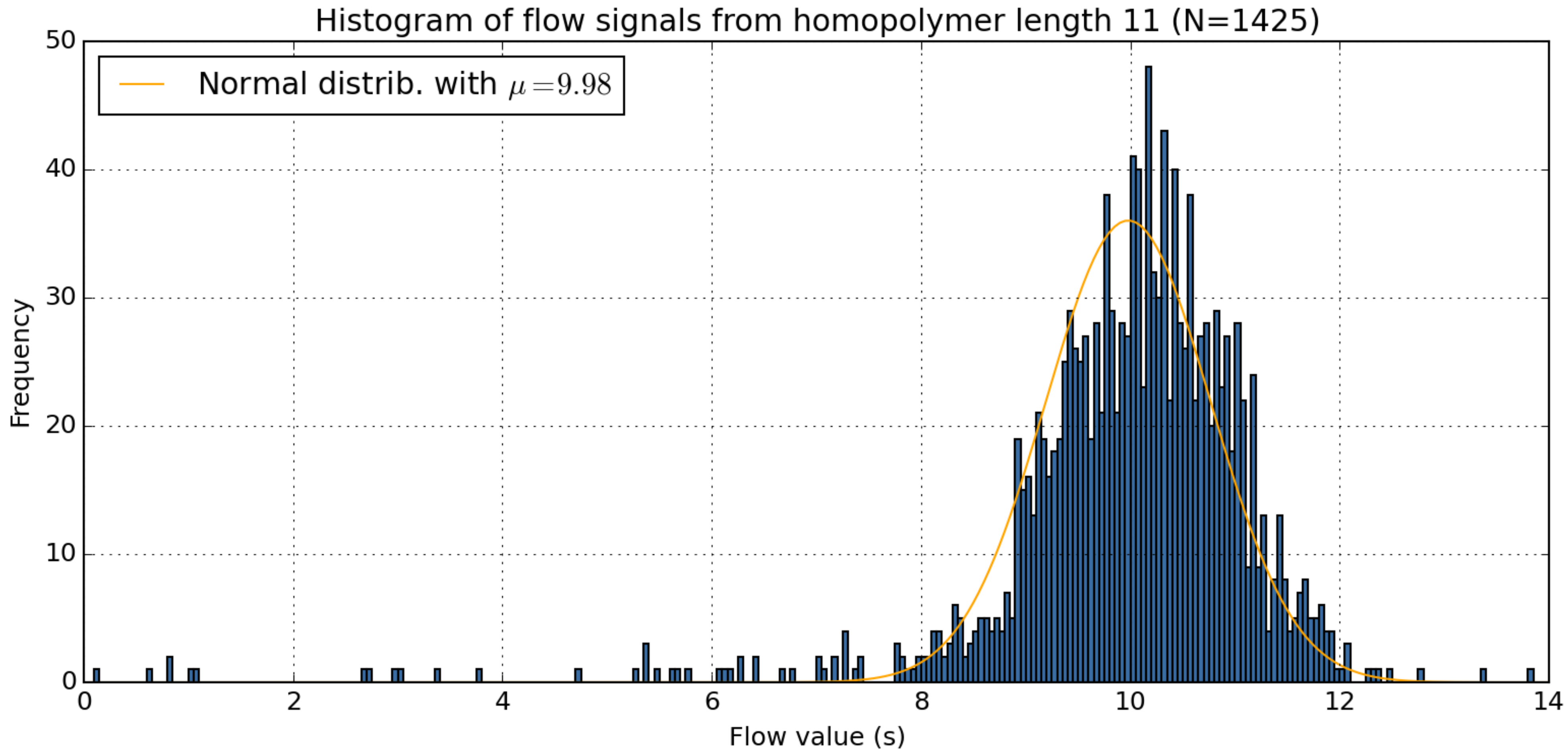

Histogram of flow signals from homopolymer length 11 (N=1425)
Normal distrib. with $\mu = 9.98$
Frequency
Flow value (s)
0
10
20
30
40
50
0
2
4
6
8
10
12
14

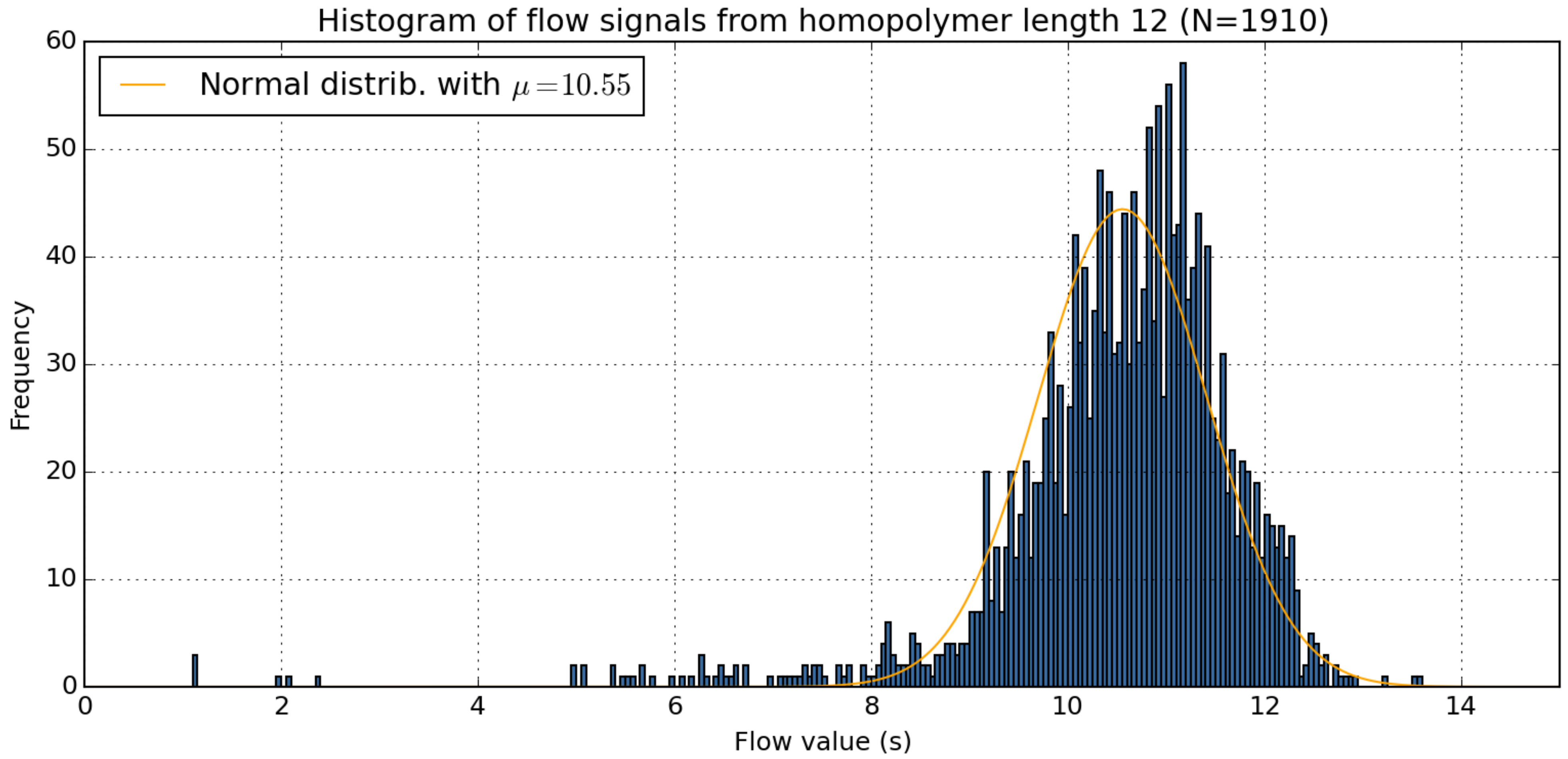
Histogram of flow signals from homopolymer length 12 (N=1910)
Normal distrib. with $\mu = 10.55$
Frequency
Flow value (s)
0
10
20
30
40
50
60
0
2
4
6
8
10
12
14

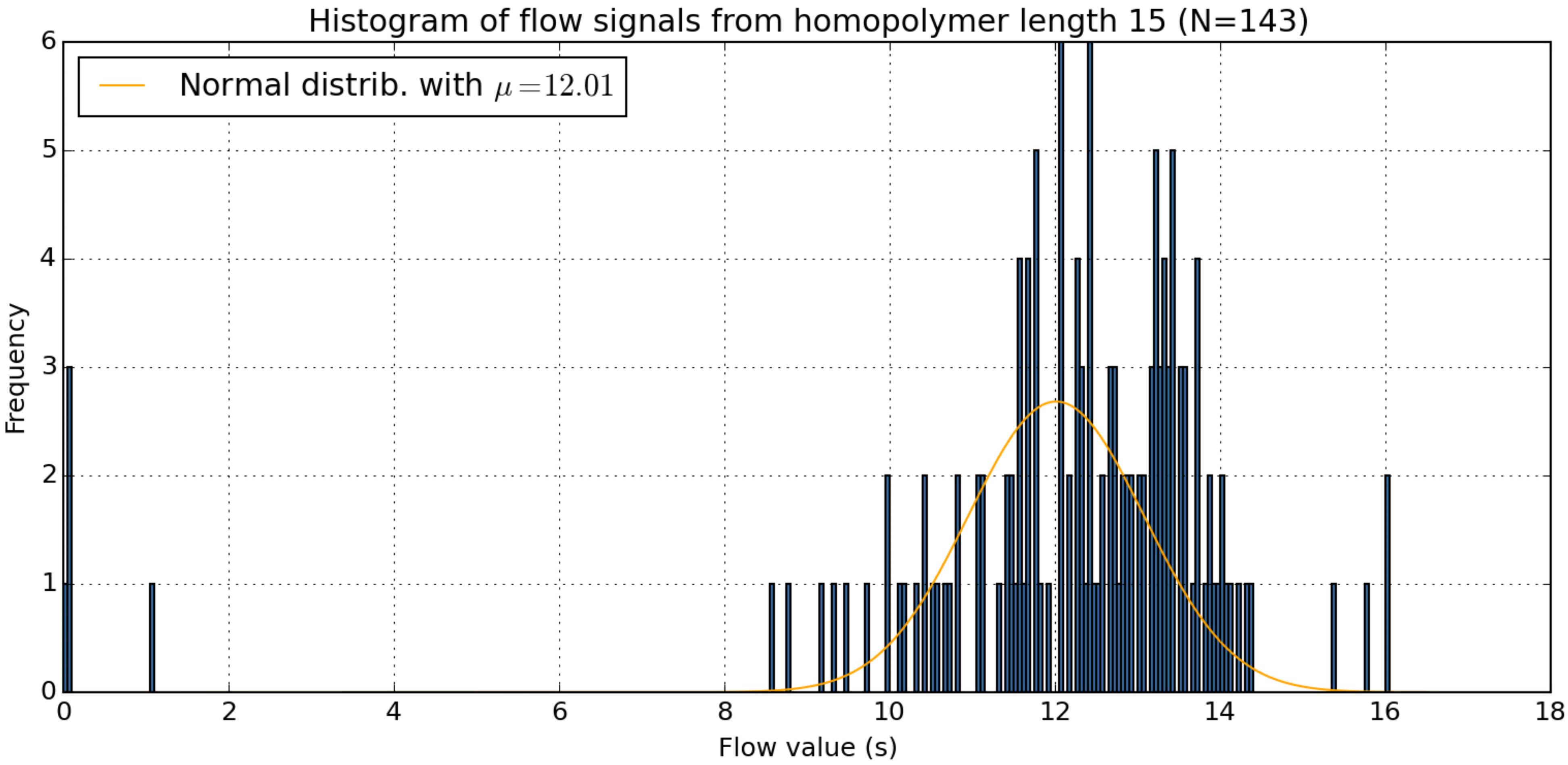
Histogram of flow signals from homopolymer length 15 (N=143)
Normal distrib. with $\mu = 12.01$
Frequency
Flow value (s)
0
1
2
3
4
5
6
0
2
4
6
8
10
12
14
16
18

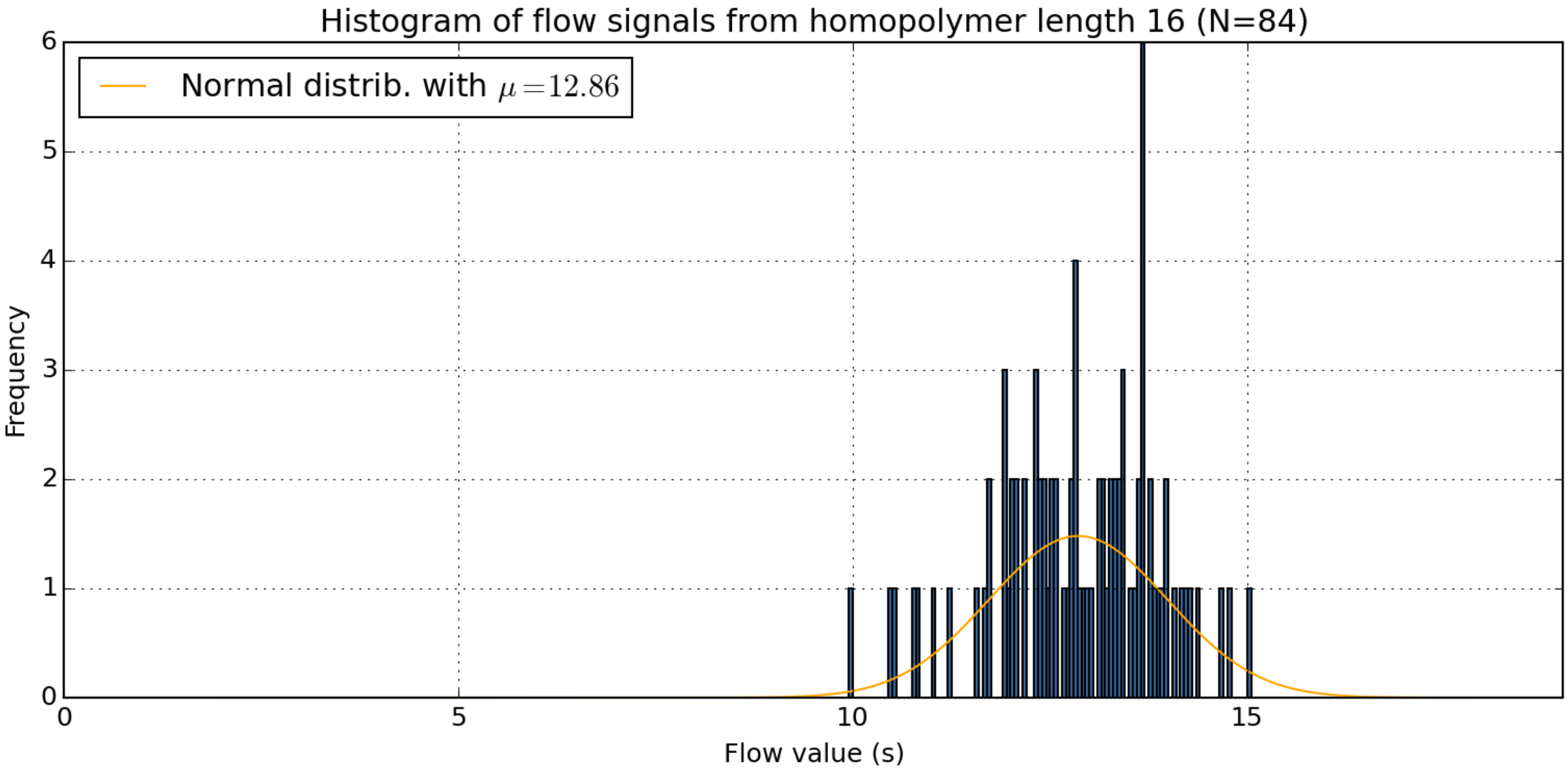

Histogram of flow signals from homopolymer length 16 (N=84)
Normal distrib. with $\mu = 12.86$
Frequency
Flow value (s)
0
5
10
15
0
1
2
3
4
5
6

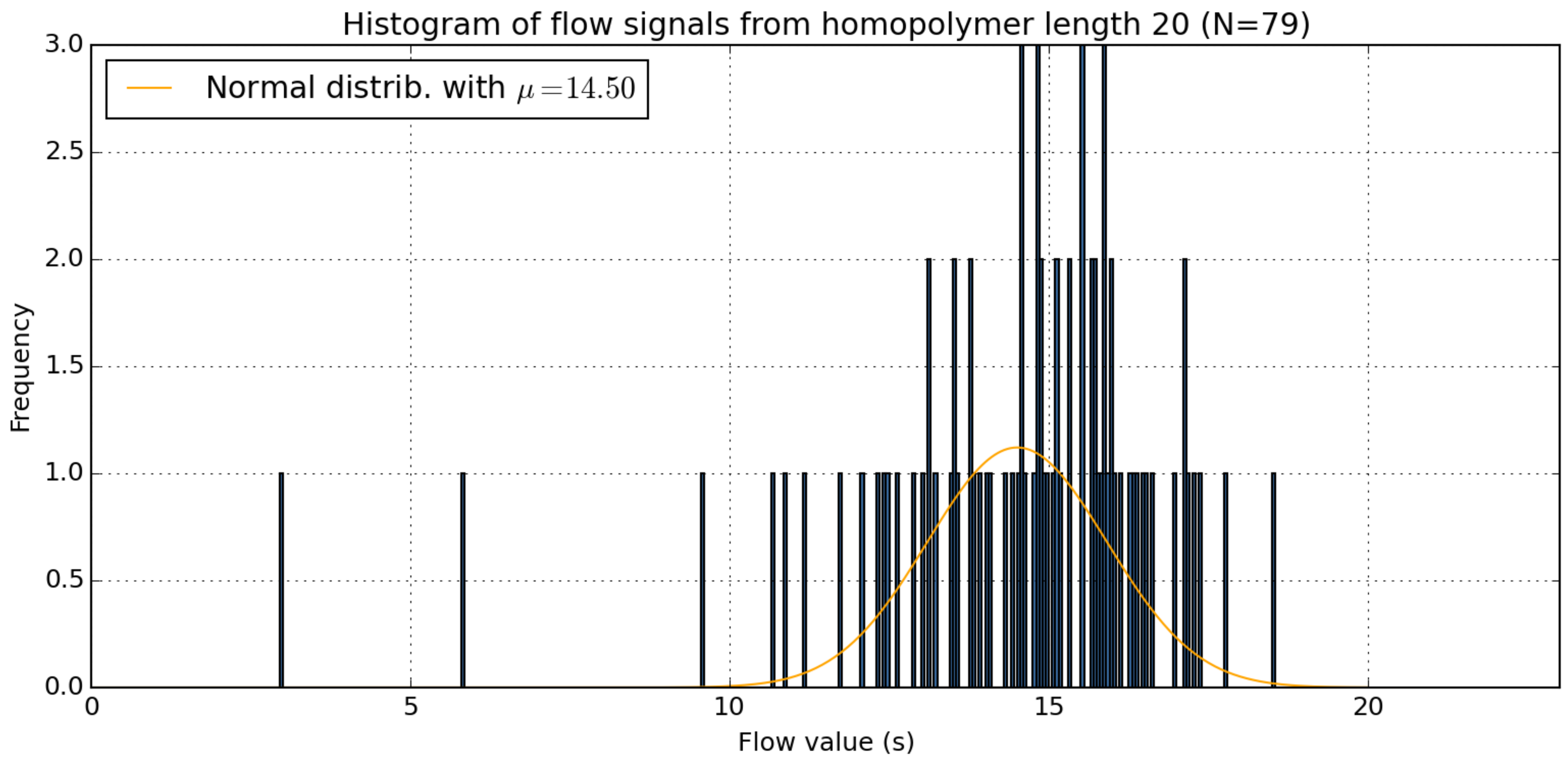
Histogram of flow signals from homopolymer length 20 (N=79)
Normal distrib. with $\mu = 14.50$
Frequency
Flow value (s)
0.0
0.5
1.0
1.5
2.0
2.5
3.0
0
5
10
15
20
